# Regression Modeling of Oxygen-Functionalized Single-Walled Carbon Nanotubes in Aqueous Dispersions

**DOI:** 10.1021/acsomega.5c00497

**Published:** 2025-08-12

**Authors:** Hoa Le, Amos Abioye, Hai V. Nguyen, Adeboye Adejare

**Affiliations:** a Philadelphia College of Pharmacy, Department of Pharmaceutical Sciences, Saint Joseph’s University, 600 S. 43rd St, Philadelphia, Pennsylvania 19104, United States; c College of Pharmacy and Health Sciences, School of Pharmacy, Belmont University, 1900 Belmont Blvd., Nashville, Tennessee 37212, United States; d Faculty of Pharmaceutical Chemistry and Technology, Hanoi University of Pharmacy, 13-15 Le Thanh Tong St., Cua Nam Ward, Hanoi 11021, Vietnam

## Abstract

Due to high drug loading and effective cell permeability, single-walled carbon nanotubes (SWCNTs) are promising nanocarriers for drug delivery systems. However, SWCNTs tend to aggregate in dispersion media because of their high aspect ratio and intrinsic hydrophobicity. Thus, the dispersion and stability of SWCNTs in water strongly depend on the dispersion method and CNT surface characteristics. Finding the optimal processing parameters and techniques for SWCNT dispersion remains challenging. This study proposes a surface modification technique (oxidation) and ultrasonication methodology to achieve well-dispersed and stable SWCNT dispersions in water. A D-optimal design was utilized to construct the experiment and investigate the effects of oxidation time and sonication conditions on hydrodynamic particle sizes (HDSs), polydispersity indices (PDIs), zeta potentials (ZPs), and Raman peak area ratios (RATs). The dynamic light scattering method was utilized to determine HDS, PDI, and ZP. Raman spectroscopy was employed to assess impurities and structural defects within SWCNTs by analyzing the Raman peak area ratio. FTIR spectroscopy and thermal gravimetric analysis were used to examine oxygenated groups on CNT surfaces. The morphology and elemental compositions of SWCNTs were determined using scanning electron microscopy combined with energy-dispersive X-ray analysis spectroscopy. The contour profiler was used to determine the optimal design space that produced dispersions with HDS, PDI, and ZP values below 250 nm, 0.350, −30 mV, respectively, and minimal defects in SWCNTs. The optimal dispersion conditions were identified using the desirability function. Overall, the models that described the relationship between the input and output factors were validated, enabling accurate prediction of responses and the optimization of input variables.

## Graphical abstract

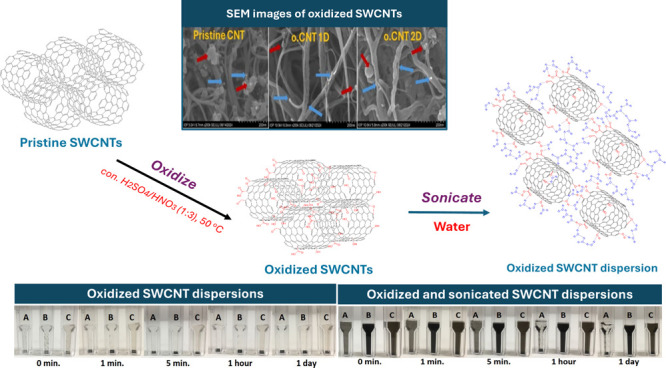

## Introduction

Carbon nanotubes (CNTs) are valuable nanocarriers for targeted and controlled drug release systems. The needle-like shape has been reported to enhance their penetration through cell membranes.^1^ CNTs have a very high surface area and an excellent drug-loading capacity along their tube length and hollow cores,^1–3^ and their surfaces are easily conjugated noncovalently or covalently with specific molecules, enabling drug delivery to targeted cells or tissues.^1–3^ CNTs are poorly dispersed in water but tend to aggregate, forming bundles or entangled ropes.^4^ Their dispersion state can change over time due to the inherent hydrophobicity and very high aspect ratios (>1000).^4–6^ Thus, mechanical dispersion and surface functionalization of CNTs are two main strategies to enhance CNT dispersibility in water.^5,7^ Ultrasonication, a mechanical dispersion technique, has been intensively used due to its simplicity and effectiveness. The hydrodynamic particle size, diameter, particle size distribution, dispersibility, and integrity of SWCNTs as a function of ultrasonic treatment time, energy, and temperature, and dispersing medium^8–16^ have been studied. Increasing sonication energy^8,9,15,17^ or sonication duration,^10,11,15,18^ decreased CNT diameter, length, and HDS have been shown to enhance the aqueous CNT dispersibility. However, oversonication may negatively affect the CNT diameter, HDS, polydispersity index, dispersibility, and integrity.^11^ Longer sonication time or higher sonication power led to more defects on CNT surfaces, including fragmented and amorphous carbon.^10,12,14,16–24^ Excessive sonication time may result in structural damage to CNTs, leading to demixing and subsequent reagglomeration into heterogeneous carbon nanotube aggregates, thereby increasing the hydrodynamic size.^22^ Therefore, it is apparent that dispersing CNTs in water by sonication is insufficient to achieve stable and well-dispersed products, especially for long-term stability, because once sonication is removed, SWCNT particles can reaggregate. In addition to the mechanical method, the functionalization of CNTs via covalent and noncovalent conjugation has been intensively used to enhance the dispersibility of CNTs in water.^5^ Oxidation of SWCNTs is the common method of covalent SWCNT surface functionalization. In the literature, various studies have investigated the oxygen functionalization of single-walled carbon nanotubes (SWCNTs) to enhance their dispersibility in aqueous media. To further improve dispersion, researchers often functionalize oxidized CNT surfaces with surfactants or polymers as dispersing agents. Combining sonication with surface functionalization is a promising approach for achieving stable and well-dispersed SWCNTs in aqueous media.^13,25–27^ Research has shown that oxidation time and sonication conditions play crucial roles in determining the characteristics of SWCNT dispersion.^28^ While these methods have been investigated independently and in combination, a gap remains in the literature regarding a systematic approach to achieving well-dispersed and stable SWCNT dispersions that minimize structural damage. To the best of our knowledge, a systematic study on the effects of these factors on the quality of SWCNT dispersion has not been reported in the literature. Additionally, finding proper sonication and oxidation conditions that balance the aqueous CNT dispersibility versus defectiveness is critical. A search through the literature reveals that no study has been reported to date to thoroughly investigate the combined effects of oxygen functionalization and sonication conditions on the stability and structural integrity of SWCNT dispersions in water. Therefore, this study addressed this gap by systematically examining the combined effects of SWCNT surface oxygenation and sonication to achieve stable and homogeneous SWCNT dispersions while preserving the structural integrity of carbon nanotubes. A D-optimal design, based on JMP Pro, was used to evaluate the effects of oxidation time, sonication power, and time on SWCNT dispersions. A mathematical model was developed to establish the relationships between these factors and HDS, PDI, ZP, and RAT. This model was then used to determine the optimal CNT dispersions with desired HDS, PDI, ZP values, and a low RAT. The SWCNT dispersions offer promising applications in drug delivery, enhancing drug solubility and permeability, facilitating controlled drug release, and targeting drugs to specific sites.

## Materials and Methods

### Materials

Single-walled carbon nanotubes (product number 698695), sulfuric acid fuming with a 20% free SO_3_ basis, hydrochloric acid (37%), and nitric acid (70%) were purchased from Sigma-Aldrich Co. (St. Louis, MO, USA). The single-walled carbon nanotubes exhibited a weight percentage of more than 70 wt % carbon basis and D × L 2–10 nm × 1–5 μm. The SnakeSkin 3.5K dialysis tubing was obtained from Thermo Fisher Scientific.

### Methods

#### Purification and Oxygen Functionalization of SWCNTs

This study used a mixture of nitric and sulfuric acids to purify and functionalize SWCNTs.^14,29–34^ The purification and functionalization of SWCNTs were carried out in three stages. Initially, pristine SWCNTs were heated to 400 °C for 4 h in the air using a furnace (Furnace 1400). Afterward, 100 mg of SWCNTs were introduced into a round flask containing 200 mL of 5 M hydrochloric acid (HCl) and stirred under magnetic stirring for 12 h. The mixture of SWCNTs and HCl was neutralized to a pH of 7 using a 5 M sodium hydroxide solution, subjected to three 30 min centrifugation cycles at 20,000 rpm, dialyzed (with dialysis tubing having a molecular weight cutoff of 3.5 kDa) for 12 h, and ultimately freeze-dried for 24 h. Subsequently, 50 mg of HCl-treated SWCNTs were dispersed into a 100 mL mixture of sulfuric and nitric acid (1/3) at 50 °C for different periods. Like SWCNTs purified with hydrochloric acid, excessive acid was removed from the oxidized SWCNTs and freeze-dried for 24 h (Figure 1). Samples were stored in a desiccator until further studies.

**Figure 1:**
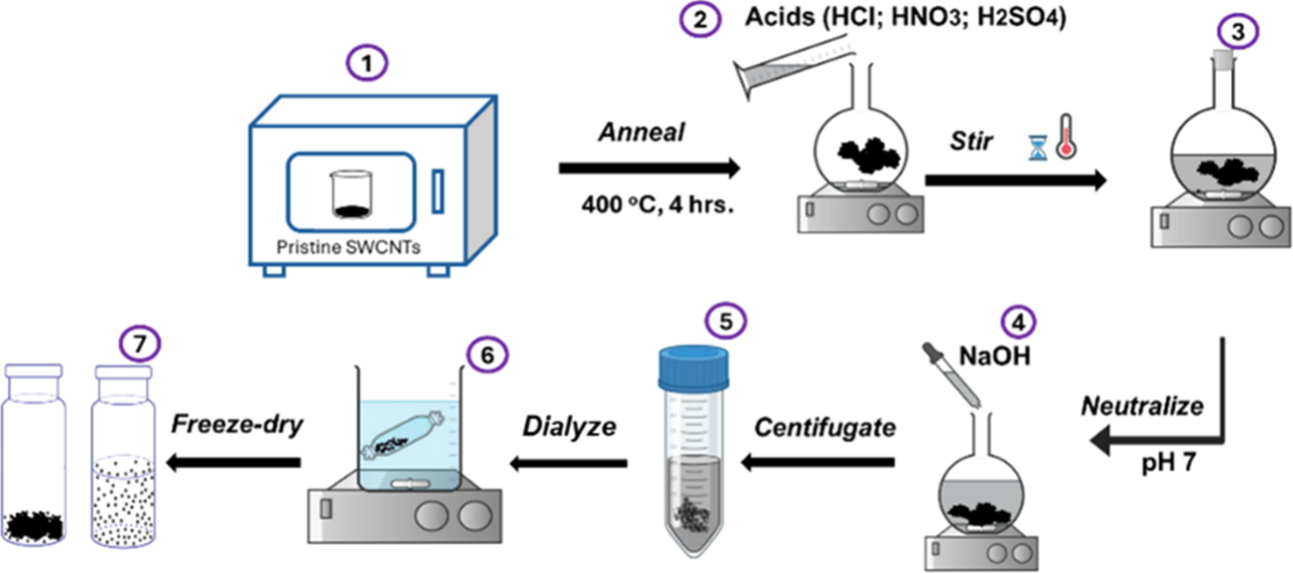
Simplified scheme for oxygen functionalization of single-walled carbon nanotubes.

#### Preparation of Aqueous SWCNT Dispersions

SWCNT dispersions were prepared by dispersing 10 mg of pristine, annealed, or hydrochloric acid-treated, or oxidized SWCNTs into 100 mL of water using a probe sonicator (Fisher Scientific Sonic Dismembrator Model 100), denoted as pristine CNT, a.CNT, and HCl_CNT or o.CNT, respectively. The samples were sonicated for different periods at various sonication power levels.

#### Characterization of SWCNT Dispersions

After purification and oxidation, the extent of disorders in CNTs was evaluated using Raman spectroscopy (with a 630 nm DeltaNu Raman spectrometer). A Thermo Scientific Nicolet FTIR spectrometer with a diamond ATR crystal was employed to assess the presence of oxygen-containing functional groups generated on SWCNT surfaces. The HDS, PDI, and ZP of SWCNT dispersions were measured using a dynamic light scattering (DLS) detector, Zetasizer Nano ZSP (Malvern, UK), with a laser incident beam at a 633 nm wavelength and a fixed scattering angle of 173◦. Thermogravimetric analysis was employed to determine the percentages of oxygen-containing functional groups in the samples before and after oxidative acid treatment, and to assess their thermal stability using a Mettler Thermogravimetric Analyzer, Model TGA/DSC 1 (Mettler Toledo). Approximately 3–5 mg of each sample was loaded into a platinum TGA pan and subsequently heated from 25 to ∼1000 °C at a rate of 3 °C/min in a nitrogen atmosphere. The morphology of SWCNTs was analyzed using Field Emission Scanning Electron Microscopy (Hitachi FE-SEM SU8100, Japan). The FE-SEM images were acquired in secondary electron and backscattering modes with an accelerating voltage of 10 kV. The elemental analysis of SWCNTs was evaluated using an energy-dispersive X-ray spectroscope (EDX) attached to the FE-SEM.

#### Experiment Design

A D-optimal design, utilizing JMP Pro software (SAS Institute, USA), was employed to screen and optimize oxidation time and sonication conditions for achieving the desired CNT dispersions. Oxidation time (X_1_), sonication power (X_2_), and sonication time (X_3_) were selected as independent variables. The HDS (Y_1_), PDI (Y_2_), ZP (Y_3_), and RAT (Y_4_) were chosen as dependent variables. The D-optimal design was used to design the experiment, identify and analyze the main, quadratic, and interaction effects, followed by modeling and optimizing the SWCNT dispersion formulations. Experimental data were subjected to standard Least Squares estimation, and a second-order polynomial relationship between the independent and dependent variables was established. The relationship is shown by the following eq (Equation 1)):

$$Y=a_{o}+a_{1}X_{1}+a_{2}X_{2}+a_{3}X_{3}+a_{12}X_{1}X_{2}+a_{13}X_{1}X_{3}+a_{23}X_{2}X_{3}+a_{11}X_{1}^{2}+a_{22}X_{2}^{2}+a_{33}X_{3}^{2}$$ 1

where Y is the predicted response value; a_o_ is the intercept term; X_1_, X_2_ and X_3_ are independent variables; *a*_1__,_ *a*_2_, and *a*_3_ are linear coefficients; *a*_12_, *a*_13_, and *a*_23_ are interaction coefficients; and *a*_11_, *a*_22_ and *a*_33_ are the quadratic coefficients.

A contour profiler based on JMP was used to find ranges of the input variables (factors) that produced desirable output factors (responses). Three optimized samples in the design space were prepared experimentally to validate the optimization, and four responses were evaluated. Desirability functionality based on JMP was used to identify optimal input variables that yielded the desired output factors. The validation of optimization was confirmed by the percentage of error and the Student *t* test. Error percentages were calculated using the following formula (eq 2).

$$Percentage\;prediction\;error(\%)=\frac{(observed\;results-predicted\;results{)}^{*}100}{observed\;results}$$ 2

## Results and Discussion

### Effects of Oxygen Functionalization and Sonication Conditions

Oxidation of CNTs has been reported to shorten their length^35^ and produce oxygen-containing functional groups on the SWCNT surface,^36–38^ which can interact with water molecules via hydrogen bonding.^32–34,39^ This interaction reduces the Val der Waal attraction between the tubes, preventing their aggregation and improving the CNT dispersibility in water. Ultrasonication involves subjecting the SWCNTs to high-frequency sound waves, which generate cavitation bubbles that implode, creating strong shear forces and disrupting the bundles.^17^ Ultrasonication can debundle CNTs by providing the necessary energy to overcome the strong van der Waals and π-π interactions between the nanotubes. As a result, the SWCNTs are dispersed as individual tubes and smaller aggregates or particles, enhancing their overall dispersibility in water.

Figure 2 presents the time-dependent stability of aqueous dispersions of (A) pristine SWCNTs, (B) SWCNTs oxidized for 1 day, and (C) SWCNTs oxidized for 2 days. The dispersions were prepared using two methods: (I) vortex mixing at 2000 rpm and (II) probe ultrasonication at 50W for 5 min. The physical stability of these dispersions was evaluated visually at room temperature at specific time intervals (0, 1, 5, and 60 min, as well as 1 day after preparation). Figure 2 (I) indicates that both oxidized and nonoxidized SWCNTs did not disperse in water, and all materials were deposited on the bottom within 1 min. This finding suggests that oxidized SWCNTs do not disperse spontaneously in water; thus, energy is required for their dispersion. As seen in Figure 2 (I) and (II), the images of the pristine SWCNT dispersions (sample A) indicate that ultrasonication successfully disrupted SWCNT aggregates and produced SWCNT dispersions. However, once the sonication was removed, noticeable reaggregation and sedimentation of SWCNTs did occur, driven by van der Waals attractions and gravitational forces. This observation suggests that ultrasonication alone was insufficient to achieve a stable and long-term dispersion.

**Figure 2:**
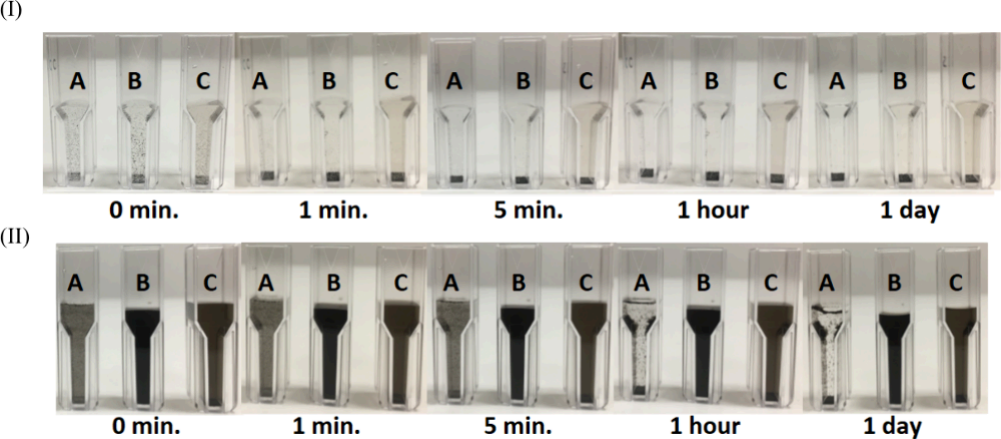
Images of aqueous dispersion stability over time of pristine SWCNTs (A) and SWCNTs oxidized for 1 day (B) and 2 days (C), prepared by (I) vortex mixing at 2000 rpm and (II) probe ultrasonication at 50W for 5 min.

Figure 2(II) shows that almost all pristine SWCNTs (sample A) were aggregated and deposited after 1 h of sonication. Additionally, oxidized SWCNT dispersions exhibited a darker and more homogeneous appearance compared to pristine SWCNTs, indicating that oxidized SWCNTs dispersed better than nonoxidized SWCNTs. These observations confirm that combining ultrasonication and oxygen functionalization can produce homogeneous/well-dispersed and stable SWCNT dispersions. However, oxidation,^30–33^ and ultrasonication^8,11,13,15^ may damage the graphitic structure and create defects on SWCNT surfaces as well as graphitic fragments and amorphous carbon, resulting in material loss and changes in CNT properties. Oxidation and sonication parameters, including oxidation time, sonication duration, and sonication power, have played critical roles in determining the quality of aqueous CNT dispersions, thus affecting their hydrodynamic diameter, homogeneity, stability, surface charge, and structural integrity.^8–10,13,14^ Therefore, in the subsequent steps, the effects of oxygen functionalization and ultrasonication on the hydrodynamic size, polydispersity index, zeta potential, and structural integrity of SWCNT dispersions were evaluated.

#### Effects of Oxygen Functionalization of SWCNTs

To investigate the effect of oxidation time on the characteristics of SWCNT dispersions, HCl_CNT was oxidized in a mixture of H_2_SO_4_/HNO_3_ (1/3) at 50 °C for varying periods (0.5, 1, 2, and 3 days), denoted as o.CNT 0D, o.CNT 0.5D, o.CNT 1D, o.CNT 2D, and o.CNT 3D, respectively. After oxidation, SWCNTs were sonicated in water at a power level of 50 W for 5 min. The HDS, PDI, ZP, and RAT of the SWCNT dispersions are presented in Table S.1 (Supporting Information). It is evident that the oxidation of SWCNTs significantly decreased the HDS, PDI, and ZP as a function of oxidation time. A longer duration of SWCNT oxidation resulted in a higher degree of oxygen-functionalization (types and amounts of oxygen-containing functional groups) on their surfaces,^36,37,40^ increasing negative-charge densities on SWCNT surfaces and reducing intermolecular interactions between SWCNTs, thereby improving dispersibility. The steady increase in absolute values of ZP from −16.90 ± 1.90 to −73.1 ± 1.48 mV from day 0 to day 3 suggests an improvement in the physical stability of the SWCNT dispersions.

The ratio of Raman peak areas calculated from the G (graphitic) and D (disordered) bands in the Raman spectrum of SWCNTs estimates the degree of disorder in carbon nanotube structures, including defectiveness, impurities, and functionalization. As seen in Figure 3, the AUC_D/G_ ratios increased steadily from 0.058 ± 0.004 on day 0 to 0.242 ± 0.010 on day 3, indicating that prolonged oxidation time increased the degree of oxygen functionalization and potential defects in the graphitic structure of SWCNTs.^35,41^ This structural damage may alter their properties, compromise the quality of SWCNT dispersions, and lead to a significant loss of CNT material.

**Figure 3:**
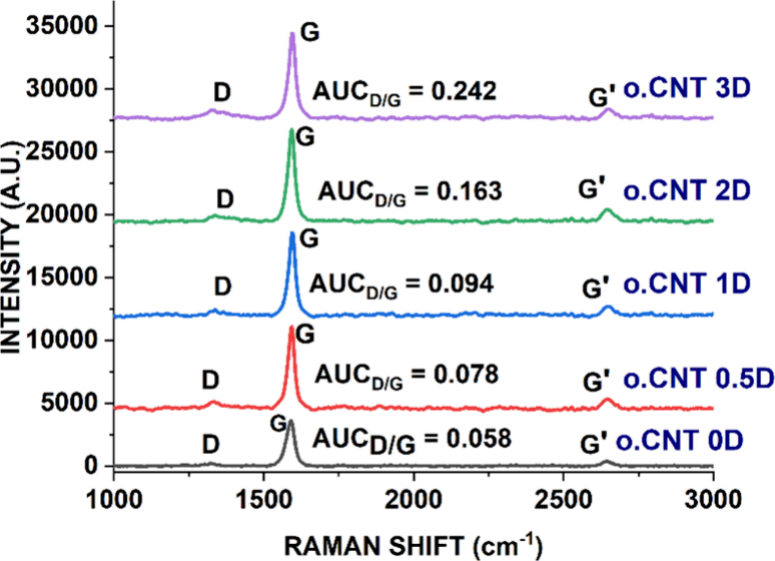
Raman spectra of pristine and oxidized SWCNTs. (HCl-treated CNTs oxidized for 0, 0.5, 1, 2, and 3 days, denoted as o.CNT 0D, o.CNT 0.5D, o.CNT 1D, o.CNT 2D, and o. CNT 3D, respectively).

The FTIR spectra of pristine and oxidized SWCNTs (Figure 4) reveal that oxidation induced the attachment of oxygen-containing functional groups to SWCNT surfaces. An increase in the intensity of the free O–H peak within the range of 3641–3660 cm^–1^, along with the broad band corresponding to hydrogen-bonded O–H groups (ranging from 3500 to 3000 cm^–1^) was observed in o.CNT 2D, o.CNT 1D and o.CNT 0.5D as the oxidation duration was prolonged. This finding suggests that an increased oxidation time, from 0.5 to 2 days, resulted in the generation of more hydroxyl groups on the CNT surfaces. Samples oxidized for 3 days (o.CNT 3D) showed a broad band ranging from 3500 to 2500 cm^–1^, assigned to hydrogen-bonded hydroxyl groups of carboxylic groups, and a band of C = O groups at 1733.44 cm^–1^, confirming the presence of carboxylic acids on SWCNT surfaces. The effect of oxidation time on the number of oxygen functional groups produced on SWCNT surfaces, the HDS, PDI, and AUC_D/G_ ratio, agrees with the findings in the literature.^42–44^

**Figure 4:**
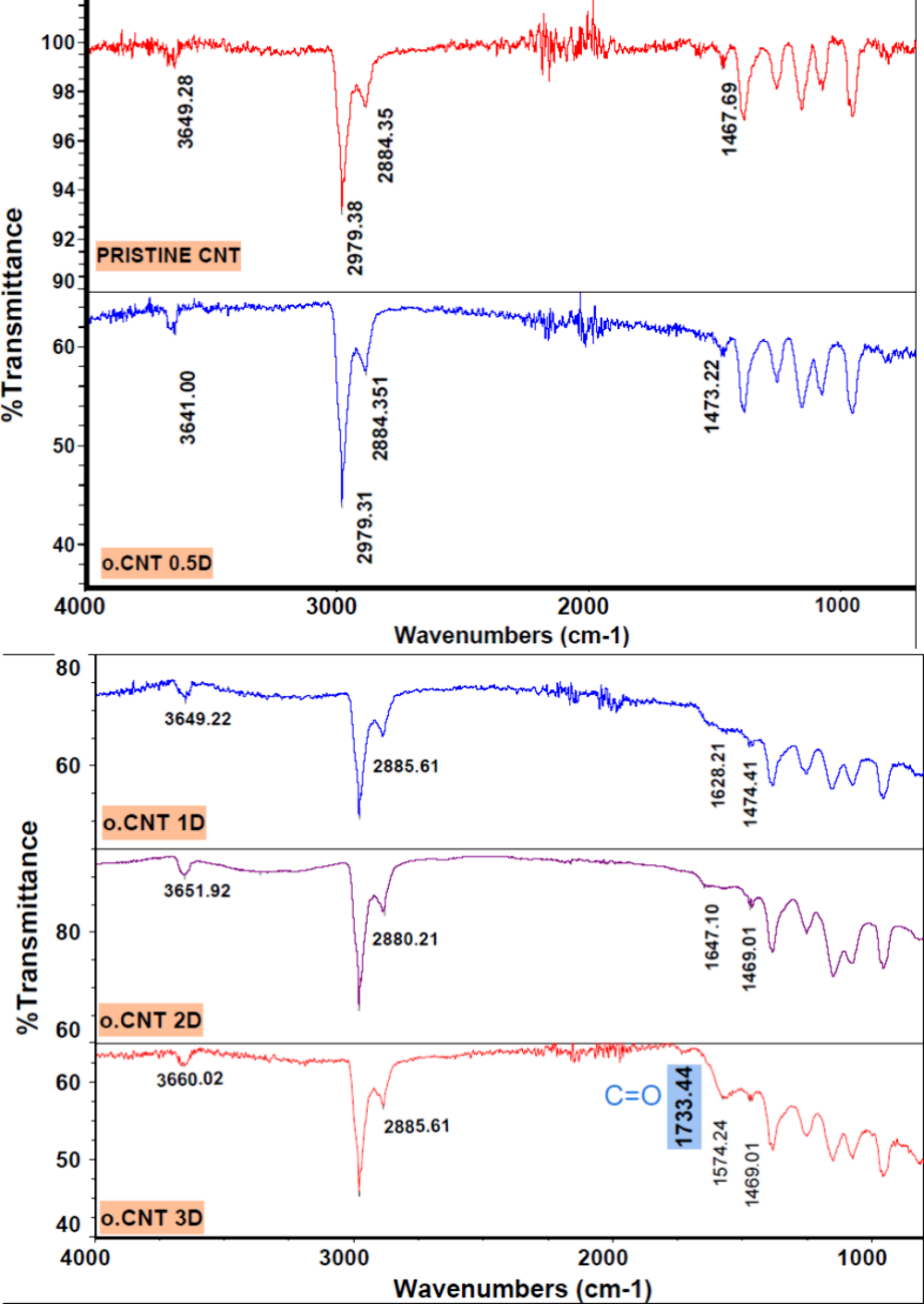
FTIR spectra of pristine and oxidized CNTs. (Raw material and HCl-treated CNTs oxidized for 0.5, 1, 2, and 3 days, denoted as pristine CNT, o.CNT 0.5D, o.CNT 1D, o.CNT 2D, and o.CNT 3D, respectively).

Figure 5 (I) and (II) show the TGA and DTG curves, respectively, of pristine CNTs and CNTs oxidized for 2 days (o.CNT 2D) and 3 days (o.CNT 3D). The thermal decomposition data of these samples are summarized in Table S.2. As seen in Figure 5 (I), the rate of weight loss was higher for o.CNT 2D and o.CNT 3D compared to the pristine CNTs, suggesting the presence of oxygen-containing functional groups after oxidative acid treatment. As seen in Figures 5 (I) and (II), the thermal degradation of CNTs occurred in several stages. The first stage, up to a temperature of 130 °C, corresponds to the evaporation of the adsorbed water. The second stage, from 130 to 350 °C, is attributed to the decarboxylation of the carboxylic groups on the CNT walls.^32^ Thermal degradation ranging from 350 to 500 °C might be due to the elimination of hydroxyl functionalities attached to the tube walls.^32^ Finally, at above 500 °C, the weight loss is associated with the decomposition of in-depth oxygen atoms tightly bonded to the remaining defective carbon atoms onto amorphous carbon^45^ and other carbonaceous impurities and CNT structures.^32^ According to the data in Table S.2, the oxygen-containing functional groups increased from 4.01% on day 0 (pristine CNT) to 14.41% on day 2 (o.CNT 2D) and 15.66% on day 3 (o.CNT 3D) of the oxidation process, confirming the successful introduction of oxygen-containing functional groups. Additionally, the weight loss of 32.24% for o.CNT 2D and 34.76% for o.CNT 3D suggests that CNT structures probably exhibited more defects as the oxidation is prolonged.

**Figure 5:**
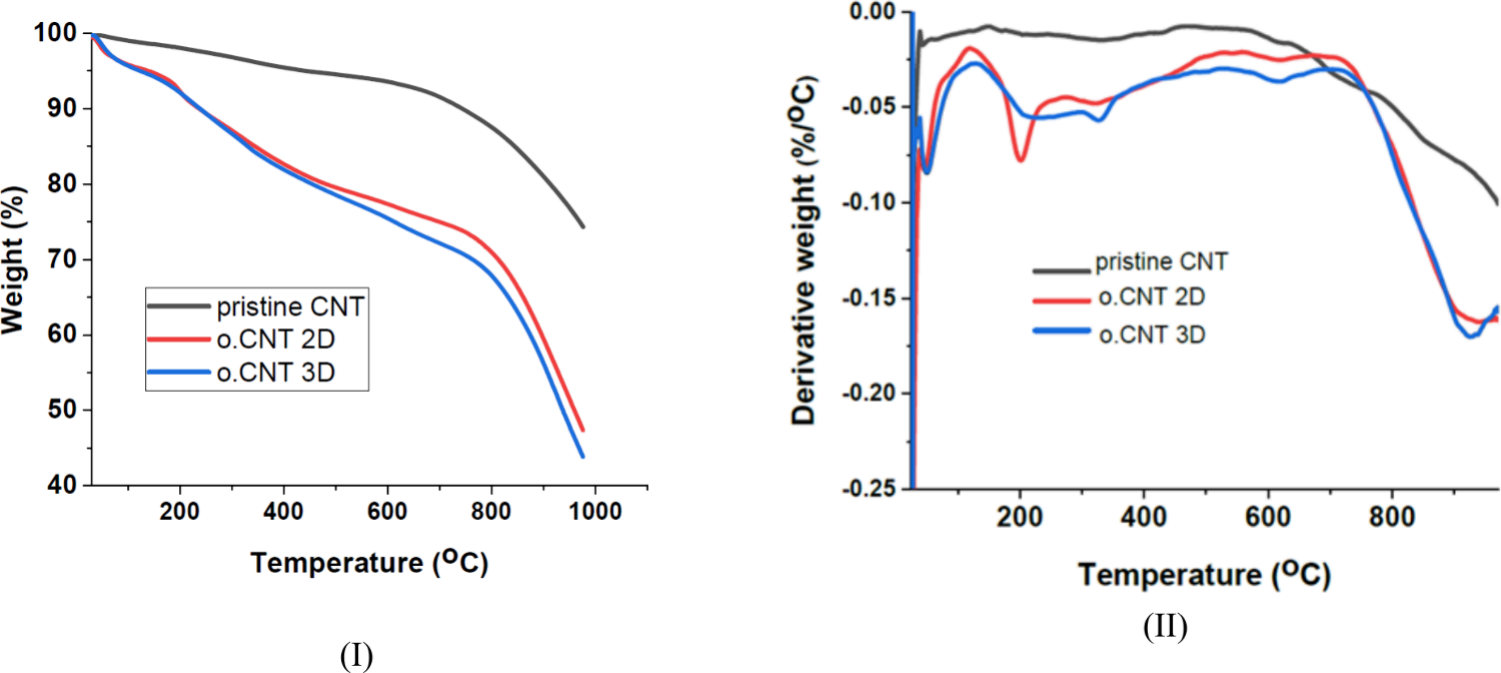
**(I)** Thermal gravimetric analysis and **(II)** derivative thermal gravimetric analysis of pristine CNTs and CNTs after oxidative acid treatment for different periods. (Raw material, HCl-treated CNTs oxidized for 2 and 3 days, denoted as pristine CNT, o.CNT 2D, o.CNT 3D, respectively).

EDX analysis and SEM images of pristine SWCNTs and purified SWCNTs are presented in Figures S.1 and 6, respectively. Air oxidation of SWCNTs is an effective and straightforward method for removing carbonaceous impurities, such as amorphous carbon, with minimal damage to the SWCNTs, thereby strengthening their three-dimensional structure. Amorphous carbon reacts with oxygen molecules in the air at 400 °C to form carbon dioxide. As seen in Figure 6, it is evident that after air oxidation, the surfaces of annealed SWCNTs (a.CNT) were cleaner and smoother than those of the pristine SWCNTs, which might be due to the removal of significant contaminants, such as amorphous carbons, from the outer wall of CNTs (identified by the red arrow). In this study, hydrochloric acid is used to remove remaining amorphous carbon and metallic catalyst impurities attached to the surfaces of SWCNTs and inside the tubes via vacancies and defect sites on the sidewalls. A decrease in the concentrations of nickel and yttrium observed in SWCNTs treated with hydrochloric acid (20.72% and 5.08% for pristine CNTs compared to 15.28% and 3.47% for HCl_CNT, respectively) was presented in Figure S.1. As seen in Figure 6, the SWCNT surfaces of the sample HCl_CNT were significantly smoother and cleaner compared to the pristine SWCNTs and the annealed SWCNTs, indicating that hydrochloric acid could remove a quantity of carbonaceous and metallic impurities from the CNT tubes (identified by the red arrow). As indicated by the blue arrows, some sidewall defects and shortened nanotubes were also observed in the HCl_CNT sample. Additionally, there was a considerable reduction in metallic content (nickel and yttrium) found in the samples treated with the HNO_3_/H_2_SO_4_ 3/1 mixture compared to those refluxed with hydrochloric acid alone (HCl_CNT). The mixture of sulfuric and nitric acids effectively facilitated the removal of residual metallic impurities in HCl_CNT. This reduction was achieved through an oxidative process that generated new defects on SWCNT surfaces, thereby providing pathways for the oxidative acids to access and dissolve the entrapped metal. A reduced amount of nickel and yttrium was observed in the sample o.CNT 3D, confirming that the SWCNTs exhibited higher purity as the oxidation duration increased. Additionally, as shown in Figure S.1, a significant increase in the oxygen content detected in SWCNTs treated with the HNO_3_/H_2_SO_4_ 3/1 mixture indicates the presence of oxygen-containing functional groups in the SWCNT structures due to these oxidative acids. The higher oxygen atomic percentages quantified with increasing oxidation durations (16.91%, 17.69%, and 20.56% for o.CNT 1D, o.CNT 2D, and o.CNT 3D, respectively) suggest that prolonged oxidation promoted the formation of additional oxygen-containing groups on SWCNT structures. Furthermore, as shown in Figure 6, after oxidative acid treatment with a mixture of HNO_3_/H_2_SO_4_ (3/1), the SWCNT surfaces became rougher and exhibited increased structural defects. The presence of shortened SWCNTs, new defects (indicated by the blue arrow), and amorphous carbon (identified by the red arrow) on the tube walls suggests that the oxidative acid mixture damaged the materials. The extended oxidation duration led to increased sidewall defects and a higher content of amorphous carbon. These EDX-SEM findings are consistent with the results obtained from FTIR, Raman spectroscopy, and TGA analyses.

**Figure 6:**
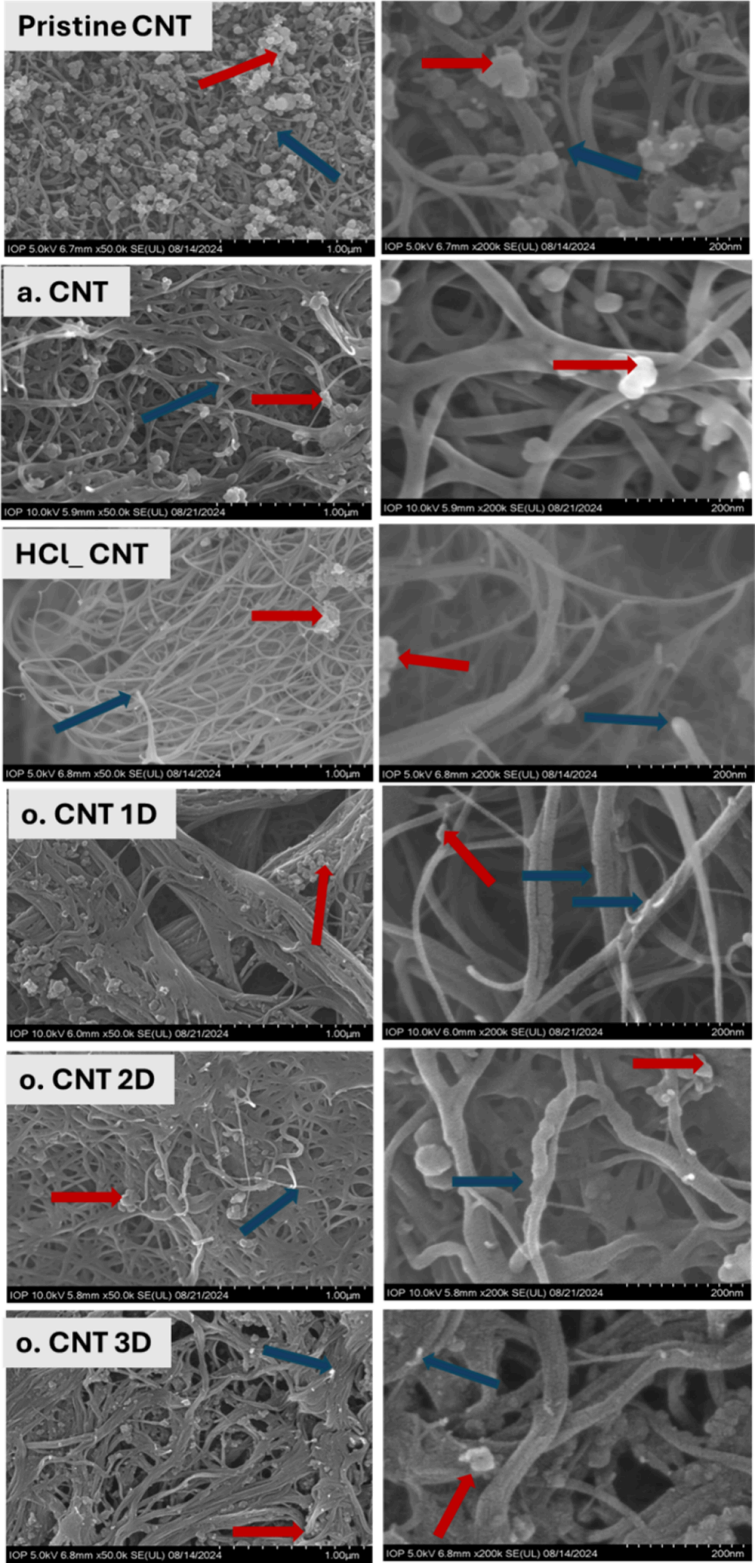
SEM images of the pristine CNTs and CNTs oxidized for different periods. (Raw material, annealed CNTs, HCl-treated CNTs, and oxidized CNTs for 0.5, 1, 2, and 3 days, denoted as pristine CNT, a.CNT, HCl_CNT, o.CNT 1D, o.CNT 2D, o.CNT 3D, respectively).

#### Effects of Ultrasonication Conditions

Sonication power determines the intensity of the sound waves applied to the dispersion, influencing the extent of aggregates/particles breakup and the quality of nanotube dispersions. To investigate the effects of sonication intensity on SWCNT dispersions, SWCNTs oxidized for 1 day (o.CNT 1D) were sonicated for 5 min at various input power levels (25, 50, 75, and 100 W). Additionally, o.CNT 1D was sonicated at a fixed input power of 50 W for different periods (3, 5, 10, and 15 min) to study the influence of sonication time on the dispersion characteristics. The HDS, PDI, ZP, and RAT of the SWCNT dispersions sonicated at different power levels and for various periods are shown in Table S.3 and Figure 7. As seen in Table S.3, increasing sonication power decreased the HDS, PDI, and ZP, suggesting that higher sonication power led to a more effective breakup of agglomerates, better dispersion, and higher negative-charge densities on SWCNT particle surfaces. Additionally, as illustrated in Figure 7, a considerable increase in the Raman peak area ratio was observed with increasing sonication power, indicating the formation of more structural defects on the SWCNTs.

**Figure 7:**
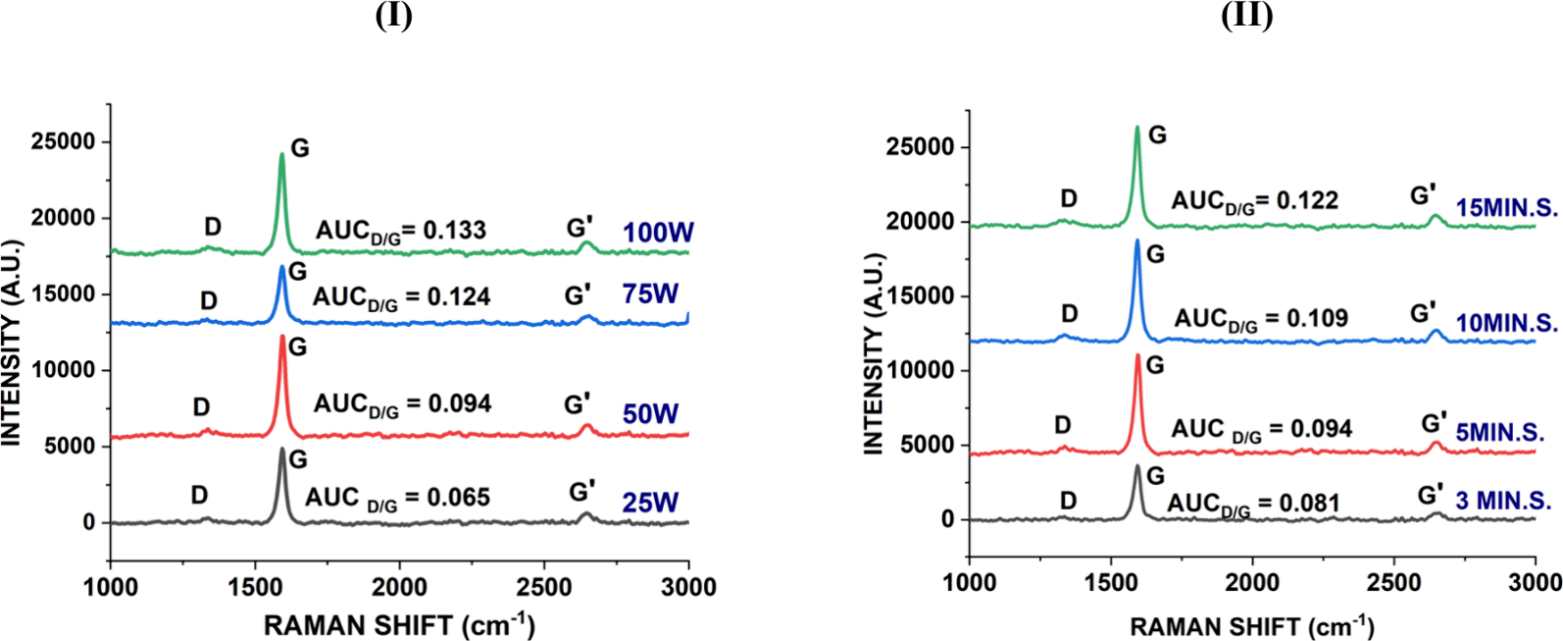
Raman peak area ratios of o.CNT_1D dispersions sonicated (I) at different sonication power levels and (II) for different periods.

The effects of sonication time on the HDS, PDI, ZP, and RAT, as shown in Table S.4 and Figure 7, exhibit the same trend as sonication power. Specifically, there was a decrease in the HDS, PDI, and ZP with increasing sonication time from 3 to 15 min, suggesting that longer sonication durations likely enhanced dispersion quality by breaking down larger agglomerates/aggregates/particles into smaller ones and promoting more uniform distribution.

Prolonged sonication increased the Raman peak area ratio, indicating that more structural defects were produced. While a short sonication period (3 to 5 min) might be insufficient to achieve good dispersions, excessive sonication could cause mechanical stress and extensive damage to the nanotubes, resulting in severe structural defects. These findings demonstrate that oxidation time, sonication power, and sonication time play crucial roles in the HDS, PDI, stability, particle surface charge, and structural integrity of SWCNTs. The interactions between oxidation time, sonication time, and sonication power are critical, as excessive power applied over a long sonication period and oxidation duration can amplify the adverse effects on structural integrity and homogeneity of CNT dispersions. Ultrasonic power and duration of exposure to ultrasound can work together, affecting the CNT diameter and length, the hydrodynamic particle size, and dispersibility.^14,25,46,47^ Therefore, it is necessary to evaluate the effects of oxidation time, sonication time, sonication power, and their interactions on CNT dispersion and structural integrity and optimize these factors to achieve an optimal, homogeneous, and stable dispersion with minimal structural defects. The following study employed a D-optimal design, generated using JMP software, to determine optimal experimental conditions.

### Experimental Design

Based on the previous study, the ranges of input factors were selected, and the responses and their limits are presented in Table 1. A total of 20 runs were performed, and the experimental results are shown in Table 2. The data are subsequently analyzed and fitted using JMP Pro 16.

**Table 1:** List of Input Factors and Their Ranges, and the List of Responses With Their Constraints for Optimization of SWCNT Dispersions

Factor	Symbol code	Experimental values			Response	Symbol code	Goal	Limits
		–1	0	1	HDS (nm)	Y_1_	Minimize	<250
Oxidation time (day)	X_1_	0.5	1.5	2.5	PDI	Y_2_	Minimize	<0.350
Sonication power (W)	X_2_	50	75	100	ZP (mV)	Y_3_	Minimize	<−30.00
Sonication time (min.)	X_3_	5	10	15	RAT	Y_4_	Minimize	<0.17

**Table 2:** Experiment Runs Designed By the JMP D-Optimal Design and Experimental Data Responses

Sample	X_1_	X_2_	X_3_	Y_1_	Y_2_	Y_3_	Y_4_
1	0.5	50	5	300.12 ± 8.07	0.448 ± 0.023	–21.15 ± 1.91	0.078 ± 0.004
2	0.5	50	10	280.08 ± 2.25	0.421 ± 0.021	–26.28 ± 1.87	0.081 ± 0.005
3	0.5	50	15	265.67 ± 2.30	0.395 ± 0.022	–30.89 ± 1.88	0.087 ± 0.006
4	0.5	75	5	281.23 ± 5.97	0.415 ± 0.019	–28.23 ± 2.01	0.095 ± 0.005
5	0.5	75	15	260.55 ± 1.89	0.375 ± 0.015	–35.51 ± 2.38	0.099 ± 0.007
6	0.5	100	5	266.38 ± 5.00	0.372 ± 0.011	–38.23 ± 1.07	0.111 ± 0.004
7	0.5	100	15	250.29 ± 1.85	0.358 ± 0.020	–40.11 ± 1.11	0.119 ± 0.006
8	1.5	50	5	200.76 ± 1.97	0.368 ± 0.006	–49.75 ± 0.94	0.127 ± 0.003
9	1.5	75	10	171.12 ± 7.98	0.329 ± 0.004	–57.22 ± 1.51	0.143 ± 0.008
10	1.5	75	10	175.61 ± 6.94	0.327 ± 0.011	–60.49 ± 0.67	0.136 ± 0.005
11	1.5	75	10	177.01 ± 4.65	0.325 ± 0.014	–59.01 ± 1.70	0.137 ± 0.006
12	1.5	100	5	185.52 ± 3.78	0.319 ± 0.004	–61.29 ± 2.22	0.149 ± 0.009
13	1.5	50	15	195.03 ± 2.67	0.336 ± 0.006	–51.56 ± 3.01	0.135 ± 0.007
14	2.5	50	5	191.59 ± 4.83	0.332 ± 0.011	–62.89 ± 2.36	0.166 ± 0.008
15	2.5	50	10	175.85 ± 6.01	0.316 ± 0.009	–68.31 ± 1.49	0.171 ± 0.005
16	2.5	50	15	185.42 ± 4.29	0.328 ± 0.005	–64.17 ± 3.28	0.176 ± 0.008
17	2.5	75	5	178.94 ± 6.11	0.323 ± 0.007	68.31 ± 1.92	0.186 ± 0.007
18	2.5	100	5	198.12 ± 2.35	0.346 ± 0.012	–60.76 ± 3.73	0.205 ± 0.009
19	2.5	100	15	235.67 ± 9.21	0.388 ± 0.014	–58.45 ± 2.56	0.212 ± 0.011
20	2.5	100	15	230 ± 5.89	0.394 ± 0.008	–59.11 ± 1.98	0.220 ± 0.008

### Model Fitting

The regression analysis of the predicted models for the HDS, PDI, ZP, and RAT is presented in Tables S.5, S.7, S.9, and S.11. The four predicted models demonstrate good fitness, evidenced by an adjusted R^2^ value of greater than 0.97 and high significance (*p* < 0.0001), with no evidence of lack of fit (*p* > 0.05). These results suggest that the models successfully express the relationship between the input factors and the responses. The coefficient estimates of the model equation for the HDS, PDI, ZP, and RAT, along with their corresponding p-values, are presented in Tables S.6, S.8, S.10, and S.12.

#### The Effects on Hydrodynamic SWCNT Particle Sizes

The HDS is significantly affected by several model terms: the intercept, X_1_, X_1_^2^, X_3_^2^, X_1_*X_2_, X_2_*X_3_, X_1_*X_3_ (see Table S.6). Additionally, the linear term of oxidation time (X_1_) has a negative effect, whereas the others (X_1_^2^, X_3_^2^, X_1_*X_2_, X_2_*X_3,_ and X_1_*X_3_) significantly positively influence this response. The other variables, sonication power (X_2_) and sonication time (X_3_), do not significantly influence this output factor. The Pareto plot (Figure S.2) illustrates the relative magnitude of the effects of the input variables on HDS, demonstrating that the most significant effect being X_1_, followed by X_1_^2^, X_1_*X_2_, X_1_*X_3_, X_2_*X_3_, X_3_^2^, X_2,_ and finally X_3_.

The model for HDS is expressed in eq 3:

$$Y_{1}=177.83-35.73\left(X_{1}-1.5\right)+13.80(\left(X_{1}-1.5\right)\left(\frac{X_{2}-75}{25}\right)+11.21(\left(X_{1}-1.5\right)\left(X_{3}-10\right)/5+6.598\left(\left(X_{2}-75\right)/25\right)\left(\left(X_{3}-10\right)/5\right)+43.97{\left(X_{1}-1.5\right)}^{2}+14.43{\left(\left(X_{3}-10\right)/5\right)}^{2}$$ 3

The response surface, prediction profiler, and interaction plots are presented in Figures 8, S.3, and S.4, respectively. It is evident that oxidation time is the most critical factor in reducing HDS. A noticeable decrease in the hydrodynamic particle size was observed with increasing oxidation duration, indicating that extended oxidation promoted size reduction. However, the particle size decreased to a minimum within the 1.5 to 2-day sonication period, followed by a steady increase with a further increase in oxidation time. It was apparent that reaggregation increased significantly with higher sonication power and more prolonged exposure periods. This observation suggests an interaction between oxidation time and processing parameters on the HDS. The surface plots (Figure 8.a) show the effects of oxidation time and sonication duration at a power level of 75 W. As the duration of oxidation and sonication increased, HDS initially decreased to a minimum, indicating that the combination of oxidation reaction time and sonication exposure time probably resulted in small particle sizes. However, further increases in these factors enlarged the hydrodynamic particle size. These findings suggest an interaction between oxidation and sonication durations on the particle size. Figure 8.a also shows that the HDS increased significantly as the SWCNTs were oxidized for more than 2.0 days, and the sonication duration exceeded 13 min. Figure 8.b shows the effect of oxidation time and sonication power on particle sizes at the fixed sonication time (10 min). Similar to the effect of sonication time, the HDS decreased significantly as oxidation duration and sonication power increased. However, intense sonication power levels (>80 W) and prolonged oxidation periods (>2.2 days) resulted in considerable enlargement of the HDS. Figure 8.c shows the effects of sonication power and sonication duration on the HDS with an oxidation duration of 1.5 days. It was observed that combining ultrasound exposure time and sonication power had a significant impact on the HDS. An increase in both parameters resulted in a reduction in the hydrodynamic particle size. Small hydrodynamic particle sizes were observed in dispersions subjected to sonication at power levels ranging from 50 to 100 W for durations of 6 to 14 min. However, exceeding 14 min of sonication resulted in a notable increase in hydrodynamic particle size. These results are consistent with Quammer et al., who showed that sonication power and duration of ultrasonication did not affect the CNT hydrodynamic diameter alone; they worked significantly in coordination.^14^ Additionally, sonication power was found to be a more significant factor than exposure periods.^8,14^ However, Yang et al. indicated that dispersibility depended on sonication energy but not the ultrasound duration or the output power of the sonicator alone.^25^

**Figure 8:**
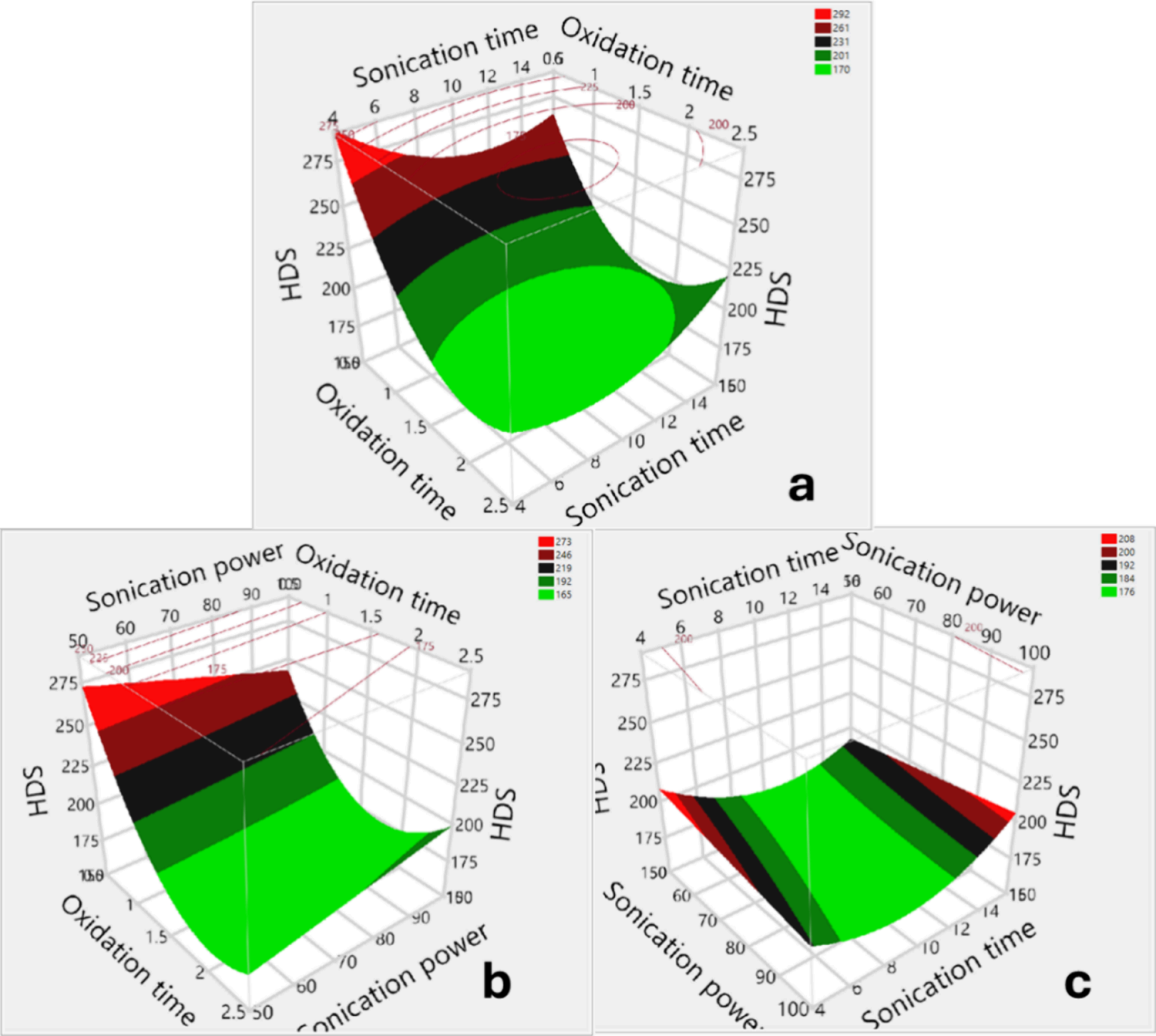
3D surface plots showing the hydrodynamic particle sizes of SWCNT dispersions as a function of: (a) oxidation time and sonication time, (b) oxidation time and sonication power, (c) sonication time and sonication power.

The above results indicate that the combination of oxidation and sonication significantly decreased the HDS. Oxidation reduced the HDS due to the breakage of the CNT structure and the shortening of their lengths. Additionally, oxidation introduced oxygen-containing groups on SWCNT surfaces, enhancing CNT dispersibility in water and preventing the formation of aggregates. Meanwhile, sonication exfoliated and shortened their length, reducing the hydrodynamic particle size. Increasing the oxidation time, sonication time, and sonication power probably induced a significant transformation of larger-diameter bundles, aggregates, or particles into smaller-diameter counterparts or individual nanotubes, thereby decreasing the hydrodynamic particle sizes. However, the interaction between two factors (oxidation time and sonication power, as well as the durations of oxidation and sonication) likely led to an increase in the hydrodynamic particle sizes (Figures 8 and S.4). Specifically, SWCNTs oxidized for an extended duration and dispersed in water at a high sonication power level or for an excessive sonication duration resulted in a significant increase in hydrodynamic particle sizes. Prolonged oxidation duration resulted in the formation of abundant oxygen functional groups that could interact with others on the particles, potentially causing reaggregation and increasing the hydrodynamic particle sizes. Prolonged oxidation periods and intense sonication likely damaged the SWCNT structures, resulting in the formation of tiny particles, individual carbon nanotubes, carbon particles, and amorphous carbon, thereby broadening the particle size distribution. Extensively damaged SWCNTs and a broadened size distribution could facilitate reaggregation, thereby increasing the HDS.^22^ Additionally, it is probable that excessive sonication duration led to demixed and damaged particles, which reaggregate into new aggregates/particles, thus increasing the HDS.^22^

#### The Effects on the Polydispersity Index

Eight model terms significantly affect the PDI, including the intercept, X_1_, X_2_, X_1_*X_2_, X_2_*X_3,_ X_1_*X_3_, X_1_^2,^ and X_3_^2^ (see Table S.8). Specifically, the model terms oxidation time (X_1_) and sonication power (X_2_) negatively affect the polydispersity index. In contrast, the other terms have a positive impact on this response. The Pareto graph (Figure S.5) illustrates the influence of these factors on the PDI, ranked in the following order: X_1_ > X_1_*X_2_ > X_1_^2^ > X_1_*X_3_ > X_2_*X_3_ > X_3_^2^ > X_2._ There is no evidence of the effects of X_3_ on the PDI. The prediction equation for the PDI is presented in eq 4.

$$Y_{2}=0.3287-0.0241\left(X_{1}-1.5\right)-0.0068\left(\frac{X_{2}-75}{25}\right)+0.0248(\left(X_{1}-1.5\right)\left(\frac{X_{2}-75}{25}\right)+0.0155\left(\left(X_{1}-1.5\right)\left(X_{3}-10\right)/5\right)+0.0121\left(\left(X_{2}-75\right)/25\right)\left(\left(X_{3}-10\right)/5\right)+0.0304{\left(X_{1}-1.5\right)}^{2}+0.0116{\left(\left(X_{3}-10\right)/5\right)}^{2}$$ 4

The response surface, prediction profiler, and interaction plots for the PDI, presented in Figures 9, S.6, and S.7, respectively, indicate that oxidation time has a significant influence on this response. As seen in Figure S.6, at low sonication power (50W) and a short sonication duration (5 min), the PDI of the dispersions noticeably decreased (from 0.456 to 0.327) as oxidation time extended from 0.5 to 2.5 days. Meanwhile, at high sonication power (100 W) and a long sonication duration (15 min), the PDI initially decreased slightly (from 0.357 to 0.341), followed by a steady increase up to 0.389 as the oxidation time increased to 2.5 days. The response surface and interaction plots (Figures 9 and S.7) indicate that two-factor interactions have a significant influence on the PDI. Specifically, PDI decreased to a minimum with increasing oxidation and sonication duration or rising oxidation time and sonication power. However, a further increase in these input parameters led to a rise in the PDI. To be specific, significant increases in the PDI were observed under the following conditions: prolonged sonication and oxidation durations (greater than 12 min and 1.75 days, respectively) (Figure 9.a) or extended oxidation time (greater than 2.20 days) combined with high sonication power (above 75 W) (Figure 9.b), or excessive sonication exposure time (more than 12.5 min) and increased sonication power (Figure 9.c).

**Figure 9:**
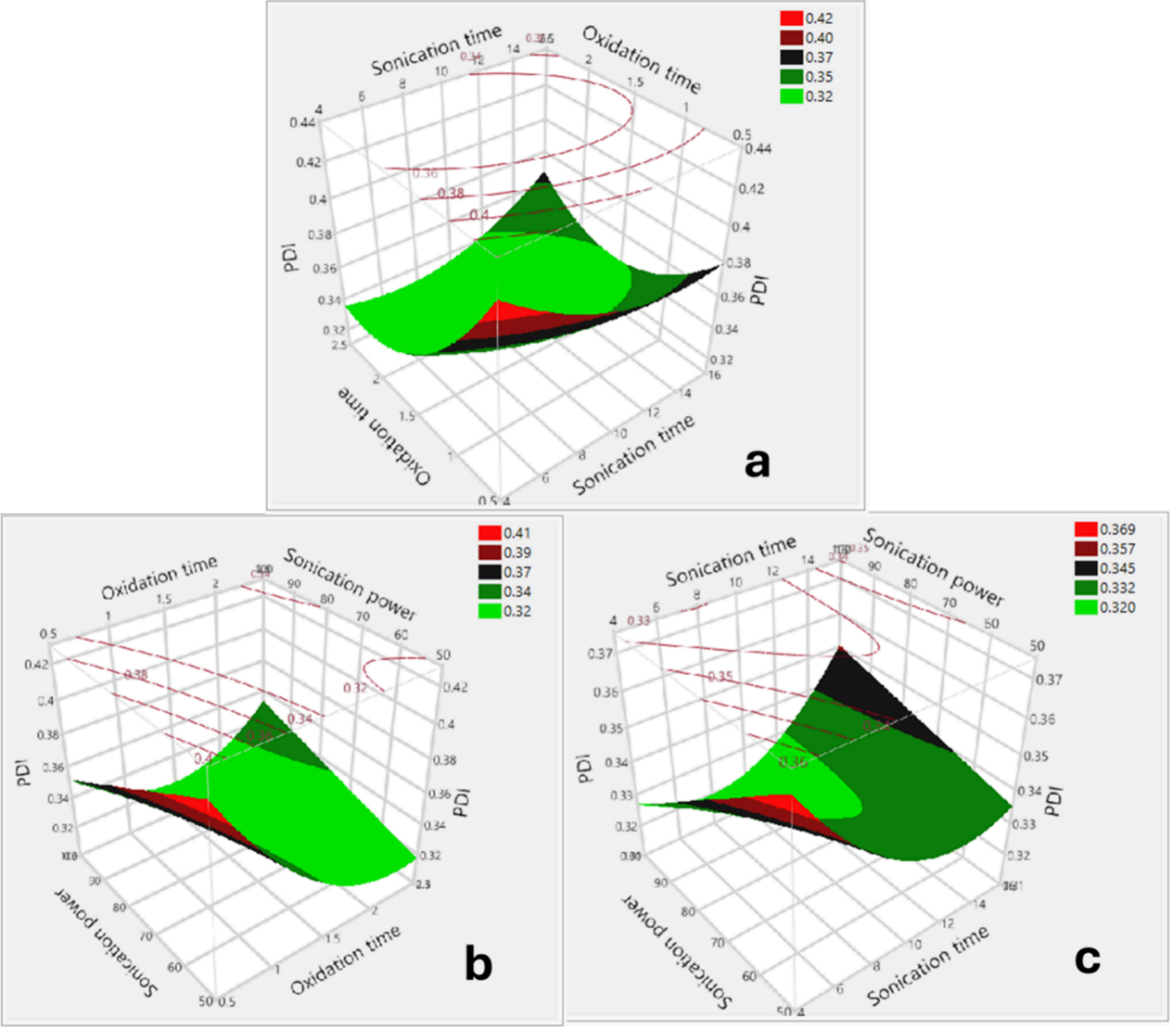
3D surface plots showing the polydispersity indices of SWCNT dispersions as a function of: (a) oxidation time and sonication time, (b) oxidation time and sonication power, (c) sonication time and sonication power.

The reduction in the polydispersity index can be interpreted in several ways. The oxidation of SWCNTs disrupted their structure and shortened their length, resulting in smaller hydrodynamic particle sizes and narrower particle size distributions. As a result, the SWCNT dispersions became more homogeneous, and the PDI decreased. Furthermore, oxidation generated oxygen-containing functional groups on SWCNT surfaces, preventing agglomeration/aggregation and improving dispersion. Concurrently, sonication exfoliated and shortened the length of SWCNTs, reducing the HDS and narrowing the particle size distribution, thus enhancing their dispersibility and contributing to a lower PDI. Extending the duration of oxidation and sonication, along with increasing the sonication power, could significantly transform the hydrodynamic particle sizes from larger aggregates and bundles into smaller particles and individual nanotubes, thereby decreasing the polydispersity index. Consequently, the combined application of oxidation and sonication effectively reduced polydispersity, enhancing the dispersibility of SWCNTs in water.

On the other hand, the increase in the polydispersity index after prolonged oxidation time may be due to the generation of numerous oxygen-containing functional groups on SWCNT surfaces and an increased interaction between the nanotubes. These alterations could lead to an increased hydrodynamic particle size, a broader size distribution, and a higher PDI. Additionally, it is most likely that using intense sonication power combined with extended oxidation periods damaged the nanotube structures, resulting in smaller tube particles, individual carbon nanotubes, carbon fragments, and amorphous carbon. Furthermore, the damaged SWCNTs and the broadened size distributions could facilitate reaggregation during sonication processing, contributing to increased HDS and PDI.^22^ Therefore, excessive sonication time could damage or demix the nanotubes into new aggregate shapes and sizes during subsequent processing, leading to increased hydrodynamic particle sizes and increased polydispersity indexes of the dispersions. These results agree with several investigations in the literature. Some authors reported that increasing sonication power led to quicker length and diameter reduction and a narrower size distribution.^8^ Yu, H., and Hermann reported that increasing the sonication time or sonication energy shortened the length of SWCNTs. They also indicated that sonication power had a greater influence on dispersion than sonication duration.^46^ Alrekabi et al. demonstrated that high-intensity sonication over short durations significantly improved CNT dispersions and exhibited less structural damage.^48^ However, Yang et al. indicated that dispersed CNTs depended on the sonication energy but not the sonication duration or the sonicator’s output power alone.^25^

#### The Effects on the Zeta Potential

Seven model terms (Table S.10) significantly affect the ZP, including the intercept, X_1_, X_2_, X_1_^2^, X_3_^2^, X_1_*X_2_, and X_1_*X_3_. Specifically, oxidation time (X_1_) and sonication power (X_2_) negatively affect the zeta potential, whereas the others have a positive influence on this response. The Pareto graph (Figure S.8) shows the impact of the input factors on the zeta potential, with their influence ranked in the following order: X_1_ > X_1_^2^> X_1_*X_2_ > X_1_*X_3_ > X_2_ > X_2_*X_3_ > X_3_^2^. There is no evidence for the influence of X_3_ and X_2_*X_3_ on this response. The correlation between X_1_, X_2_, and X_3_ on the zeta potential is defined in eq 5.

$$Y_{3}=-58.63-15.39\left(X_{1}-1.5\right)-3.12\left(\frac{X_{2}-75}{25}\right)+4.48(\left(X_{1}-1.5\right)\left(\frac{X_{2}-75}{25}\right)+2.20\left(\frac{\left(X_{1}-1.5\right)\left(X_{3}-10\right)}{5}\right)+7.79{\left(X_{1}-1.5\right)}^{2}+3.36{\left(\left(X_{3}-10\right)/5\right)}^{2}$$ 5

The response surface and prediction plots (Figures 10 and S.9) indicate that oxidation time has a significant impact on the zeta potential (ZP), followed by sonication power. As the oxidation duration increased, the ZP decreased significantly, which suggests that the surface charges became more negative, indicating increased physical stability of the SWCNT dispersions. Additionally, there was a moderate reduction in the ZP as sonication power increased. Figures 10.a and S.10 illustrate the interactions between oxidation time and sonication power on the ZP. Specifically, as shown in Figure 10.a, at low sonication power and an extended oxidation time, a decrease in the ZP was observed. However, under high sonication power, the ZP initially decreased with increasing oxidation time, followed by a very slight increase as both parameters continued to rise. The interaction plots (Figure S.10) demonstrate that at high sonication power (100 W) and an extended oxidation time (greater than 2.2 days), the zeta potential values increase moderately (indicating low surface charge densities), which suggests less physical stability.

**Figure 10:**
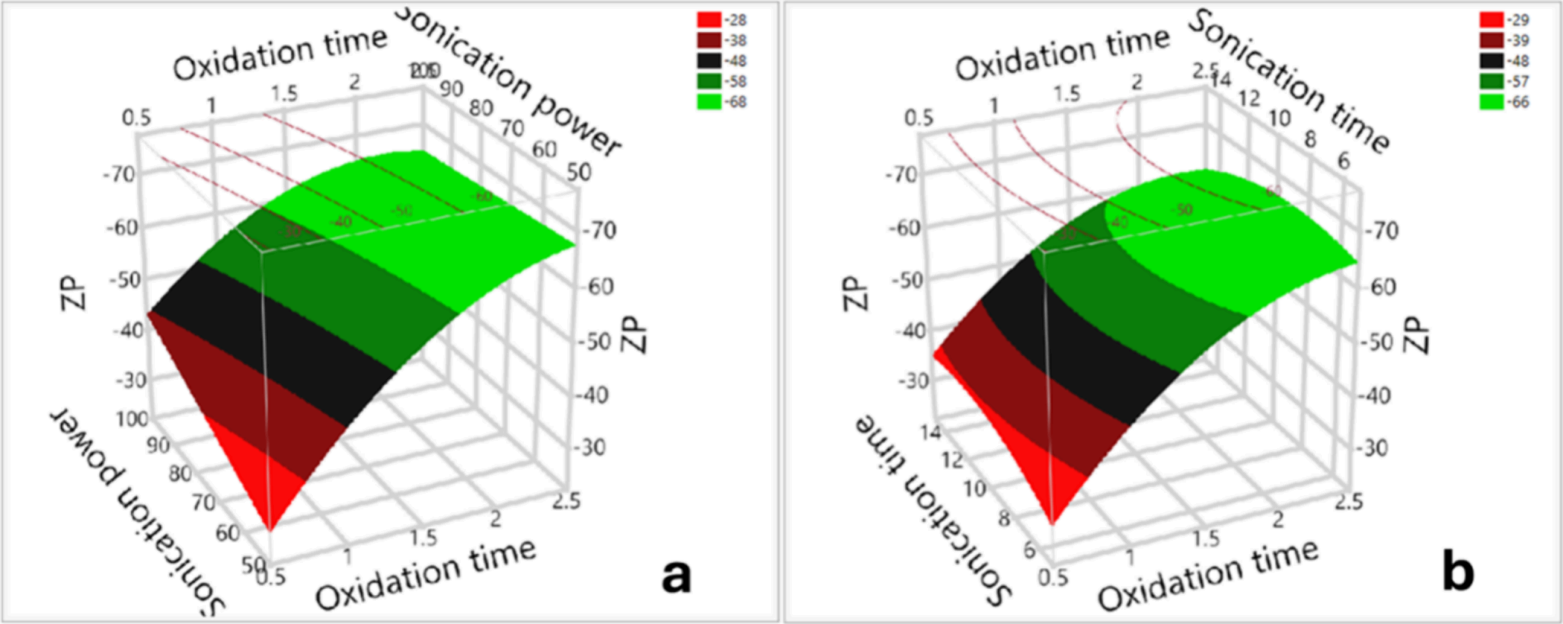
3D surface plots showing the zeta potentials of SWCNT dispersions as a function of (a) oxidation time and sonication power, (b) oxidation time and sonication time.

Figure 10.b shows that as the oxidation duration increased, the ZP decreased significantly; in contrast, with an extended sonication time, the ZP remained unchanged. As seen in Figures 10.b and S.10, there was an interaction between the oxidation and sonication time on the ZP. With extended durations of oxidation and sonication, the zeta potential initially declined, followed by a slight rise upon continued processing. Specifically, with prolonged oxidation time (greater than 1.2 days) and a rise in sonication time (greater than 10 min), the zeta potentials increased moderately (Figure 10.b).

The observations in Figures 10, S.9, and S.10 can be interpreted in several ways. SWCNT oxidation and the reduction in hydrodynamic particle sizes decreased the ZP. The oxidation of SWCNTs introduced oxygen-containing functional groups on their surfaces, leading to negatively charged particle surfaces.

SWCNTs oxidized for extended oxidation periods exhibited numerous oxygen-containing groups, increasing the negative charge density on the surfaces of the particles, and, therefore, the zeta potential values decreased. Furthermore, oxidation and sonication power broke up aggregates, reducing the hydrodynamic particle sizes and improving the dispersibility of SWCNTs. Consequently, the overall surface areas between particles and the dispersion medium (water) could be enhanced, leading to enhanced exposure of negatively charged functional groups on the particle surfaces. Hence, the zeta potential values of the SWCNT dispersions decreased. However, prolonged oxidation duration, intense sonication power, and excessive sonication time increased zeta potential values. These findings can be explained as follows. It is most likely that an intense sonication power level and a prolonged oxidation period damaged tube structures and broadened the size distribution, thereby facilitating reaggregation. Additionally, with extended sonication exposure, SWCNTs could be damaged or demixed and reaggregated into new agglomerate sizes during subsequent processing, increasing the HDS.^22^ The aggregation might reduce the overall surface area between SWCNTs and water, hindering the exposure of negatively charged groups on the particle surfaces. Consequently, the density of surface negative charges decreased, resulting in increased zeta potential values.

#### The Effects on the Raman Peak Area Ratio

Four model terms (Table S.12) indicate what significantly affects the Raman peak area ratio, including the intercept, oxidation time (X_1_), sonication power (X_2_)_,_ and sonication time (X_3_). Two-factor interaction terms and quadratic terms have no significant effect. The model for this response is expressed in eq 6.

$$Y_{4}=0.1395-0.0465\left(X_{1}-1.5\right)-0.0162\left(\frac{X_{2}-75}{25}\right)+0.0049\left(X_{3}-10\right)/5)$$ 6

The Pareto plot (Figure S.11) shows the effects of the independent variables on the Raman peak area ratio from the most significant to the smallest. The impact of the factors is arranged in the following order: X_1_ > X_2_ > X_3_.

Response surface, prediction profiler, and interaction plots visually display the impacts of oxidation time, sonication time, and sonication power on Raman peak area ratios, as shown in Figures 11, S.12, and S.13, respectively. These figures demonstrate that prolonged oxidation and sonication durations, along with higher sonication power, contributed to an increase in the Raman peak area ratio. It is well-known that the oxidation of single-walled carbon nanotubes increases RAT by producing defects, functional groups, small graphitic fragments, carbon fragments, and amorphous carbon. Sonication can also exfoliate and fragment nanotubes, producing surface defects, small CNT fragments, carbon particles, amorphous carbon, and structural alterations, which in turn lead to increased Raman peak area ratios. Clearly, oxidation time had a significant influence on the ratios, followed by sonication power and, subsequently, sonication time (see Figure S.12). These findings suggest that intense ultrasonication resulted in surface defects and severe damage to the tube structures. Sonication durations longer than the optimum caused severe damage to the nanotubes. Sonication power is more critical than sonication time in breaking up and shortening the tube length.^8,14^ Figures 11 and S.13 indicate no significant two-factor interaction between oxidation time, sonication time, and sonication power on the Raman peak area ratios.

**Figure 11:**
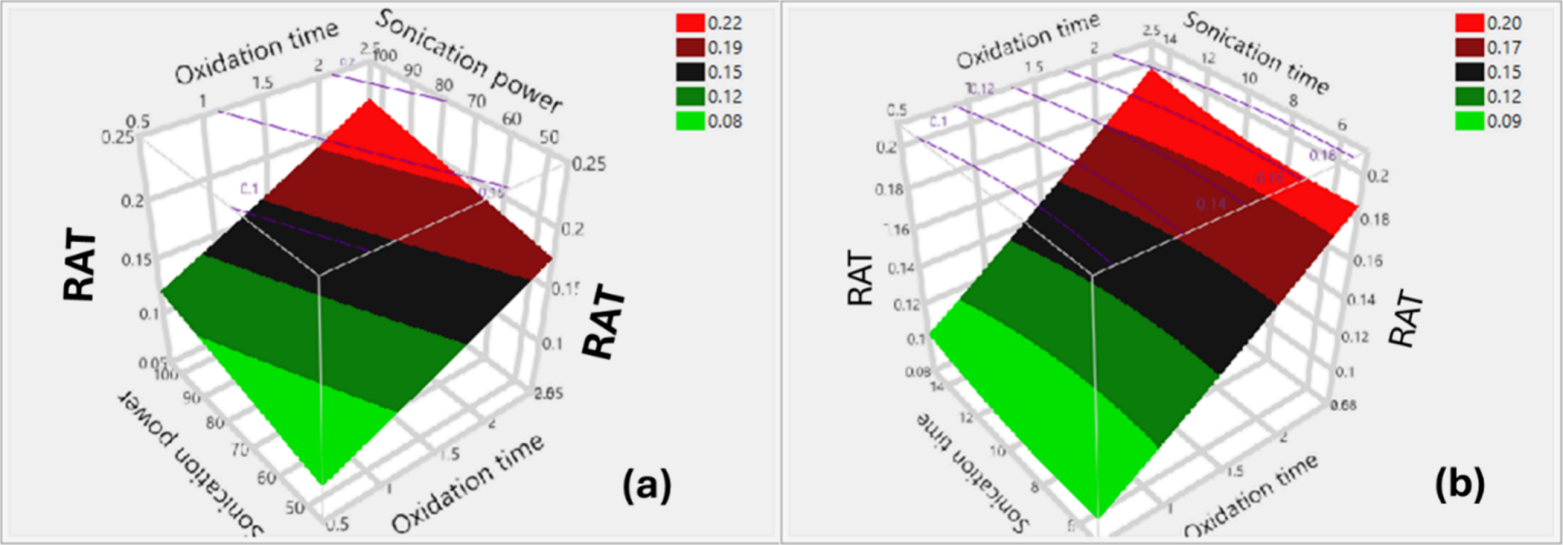
3D surface plots showing the Raman peak area ratios of SWCNT dispersions as a function of: (a) oxidation time and sonication power, (b) oxidation time and sonication time.

The results above show that the models adequately represent the relationships between the input factors (oxidation time, sonication time, and sonication power) and the responses (HDS, PDI, ZP, and RAT). In the next step, optimization tools based on JMP Pro 16 utilize these equations to identify ranges of the input variables that yield desirable responses.

### Optimization of Aqueous SWCNT Dispersions

The ultimate objective of this study is to obtain stable and well-dispersed SWCNT dispersions in an aqueous medium. Optimizing input factors (oxidation time, sonication time, and sonication power) to achieve small hydrodynamic particle sizes and polydispersity indexes, high absolute zeta potential values, and minimal Raman peak area ratios is crucial for producing SWCNT dispersions with high stability and minimal damage, which are essential characteristics for effective drug delivery.

As drug delivery systems, SWCNT dispersions must be well-dispersed and stable in water, as the stability of these dispersions influences the uniformity of the drug within the dispersion, which in turn affects both therapeutic efficacy and drug safety. The hydrodynamic size, polydispersity index, and zeta potential are critical indicators of the physical stability of single-walled carbon nanotube dispersions in aqueous media. A dispersion is considered highly monodisperse, moderately or highly polydisperse based on the PDI ≤ 0.1, ranging from 0.1 to 0.4 or ≥ 0.4, respectively.^49^ A dispersion with zeta potential values >30 mV or < −30 mV is considered highly stable.^49,50^

As drug delivery carriers, SWCNTs can entrap drugs onto their surfaces and/or within their internal structure. Thus, maintaining the structural integrity of SWCNTs is essential for efficient drug loading and controlled release. The Raman peak area ratio reveals the structural integrity of SWCNTs and the functionalization on their surface. Therefore, SWCNTs used for drug delivery should exhibit minimal damage, indicated by a low Raman peak area ratio, to ensure effective drug loading and controlled release capabilities. Currently, our lab is evaluating drug-loaded oxygen-functionalized SWCNTs, and the findings will be published separately.

This study utilizes the contour profiler and desirability function to determine the optimal conditions for input factors, which are constructed based on the desired responses (listed in Table S.13). The desirability function is employed to find the optimum parameters based on specific targets and response limits for each response. The desirability for all responses is identified as the geometric average of the desirability functions for the individual responses. The prediction profiler (Figure S.14) displays the predicted response at specified values of each variable: oxidation time, sonication power, and sonication time. The highest desirability (0.76) is achieved when SWCNTs oxidized for 2 days and sonicated for 10 min at a power of 50 W (Table S.13). Under these optimal conditions, SWCNT dispersion exhibited the predicted values for the HDS, PDI, ZP, and RAT with 165.4 nm, 0.319, −63.49, and 0.146, respectively (Table S.13 and Figure S.14).

The space design aims to determine the range of acceptable parameters or formulas that yield the desired responses. The contour profiler represents the design space for input factors (oxidation time, sonication energy, and sonication time), with output factors including HDS, PDI, ZP, and RAT, as shown in Figure S.15.

### Optimization Process Validation and Model Verification

Finally, the validation of the optimization process and model verification was evaluated. Specifically, three optimal dispersions (three points) in the design space, as determined using the contour profiler, and three optimal SWCNT dispersions, as determined using the desirability function, were experimentally conducted. Four responses were determined. The optimization process, validation, and model verification were evaluated. The optimal parameters for input factors and predicted responses of the dispersions using the desirability function and their observed responses, error percentages, and p-values are presented in Tables S.13 and S.14, respectively. Similarly, the predicted values, observed values, error percentages, and p-values of the dispersions using the design space are also shown in Table S.15.

The data (Tables S.14 and S.15) indicate that the observed values match the predicted values well, with low error percentages (below 5%). Additionally, the p-values reported from the Student *t* test are higher than 0.05, indicating that the differences between observed and predicted values are insignificant. It demonstrates that the models are successful in prediction and can be used to predict responses and optimize input factors. Figures S.16 and S.17 display the particle characteristics of pristine SWCNT dispersion and optimal SWCNT dispersion, respectively, using the desirability function.

## Conclusion

This study successfully developed SWCNT aqueous dispersions using the combination of SWCNT oxidation and ultrasonication. A D-optimal design was generated using JMP software to understand the effect and the interaction between the input and output factors. We found that oxidation duration played the most crucial role in the aqueous dispersion of SWCNTs, followed by sonication power and then sonication time. Oxidation and sonication reduced the hydrodynamic particle sizes, improving their dispersibility and physical stability. However, excessive oxidation and sonication durations, along with intense sonication power, led to increased hydrodynamic particle size due to potential reaggregation. We successfully applied a multiple linear regression model to optimize the factors of oxidation duration, sonication time, and sonication power for producing desirable single-walled carbon nanotube (SWCNT) dispersions. The models were validated to demonstrate their ability to accurately predict responses and optimize input parameters. The design space and the optimal parameters were identified. The obtained dispersions met the requirements for oral drug delivery systems in terms of size range, polydispersity index, zeta potential, and minimized structural defects. These optimized SWCNT dispersions have potential applications in oral drug delivery, including enhancing drug solubility and permeability, controlling drug release, and targeting drugs to specific sites.

## Supplementary Material



## Data Availability

All data generated or analyzed during this study are included in this published article and its Supporting Information files.

## Notes

Physicochemical properties of oxidized and sonicated SWCNT dispersions, as well as the results of second-order polynomial regression modeling, optimization, and validation of the optimal dispersions; relative content of oxygen-containing functional groups and the elemental composition of pristine SWCNTs and samples oxidized for different durations; characteristics of SWCNT dispersions using CNTs oxidized for different periods (Table S1); weight loss of oxidized SWCNTs (Table S2); characteristics of CNT dispersions sonicated at different sonication power levels (Table S3); characteristics of SWCNT dispersions sonicated for various periods (Table S4); regression analysis of predicted models for the HDS (Table S5); regression coefficients and *p* values calculated from the HDS model (Table S6); regression analysis of predicted models for the PDI (Table S7); regression coefficients and *p* values calculated from the PDI model (Table S8); regression analysis of predicted models for the ZP (Table S9); regression coefficients and *p* values calculated from the ZP model (Table S10); regression analysis of predicted models for the RAT (Table S11). regression coefficients and *p* values calculated from the RAT model (Table S12); optimal parameters for input factors and predicted responses of the SWCNT dispersion using the desirability function (Table S13); observed responses of the optimal SWCNT dispersions using the desirability function and results of the optimization process validation (Table S14); validation results of the optimization process using the contour profiler (Table S15); EDX spectra and elemental compositions of pristine SWCNTs and SWCNTs oxidized for different days (Figure S1); Pareto plot analysis for the contribution of factors affecting HDS (Figure S2); prediction profiler plots for the HDS of aqueous SWCNT dispersions (Figure S4); two-factor interaction plots for the HDS of aqueous SWCNT dispersions (Figure S5); Pareto plot analysis for the contribution of input factors affecting PDI (Figure S5); prediction profiler plots for the PDI of aqueous SWCNT dispersions (Figure S6); two-factor interaction plots for the PDI of aqueous SWCNT dispersions (Figure S7); Pareto plot analysis for the contribution of factors affecting ZP (Figure S8); prediction profiler for the ZP of aqueous SWCNT dispersions (Figure S9); two-factor interaction plots for the ZP of aqueous SWCNT dispersions (Figure S10); Pareto plot analysis for the contribution of factors affecting RAT (Figure S11); prediction profiler for the RAT of aqueous SWCNT dispersions (Figure S12); two-factor interaction plots for the RAT of aqueous SWCNT dispersions (Figure S13); prediction profiler showing the optimized SWCNT dispersion with maximum desirability (Figure S14); contour profiler output showing the design space of aqueous SWCNT dispersions (Figure S15); hydrodynamic particle size distribution and zeta potential of the pristine and the optimal SWCNT dispersions (Figure S16 and S17, respectively) (PDF)

## b

Department of Pharmaceutical Industry, Hanoi University of Pharmacy, 13–15 Le Thanh Tong St., Cua Nam Ward, Hanoi 11021, Vietnam
