# Supplementary material for: Regression Modeling of Oxygen-Functionalized Single-Walled Carbon Nanotubes in Aqueous Dispersions
Source: ACS Omega. 2025 Aug 12;10(33):37066–80. doi: 10.1021/acsomega.5c00497 (PMC12391941; doi:10.1021/acsomega.5c00497)
Supplement: Supplementary file 1 [file ao5c00497_si_001.pdf]

Regression modeling of oxygen-functionalized single-walled carbon nanotubes in aqueous dispersions

Hoa Le [a, b], Amos Abioye [c], Hai V. Nguyen [d], and Adeboye Adejare [a*]

[*] Corresponding Author:

Adeboye Adejare, Ph.D.:

[a] Saint Joseph's University, Philadelphia College of Pharmacy, Department of Pharmaceutical Sciences, 600 S. 43rd St, Philadelphia, Pennsylvania 19104, United States;

Email: aadejare@sju.edu

Authors:

- Hoa Le:

  [a] Saint Joseph's University, Philadelphia College of Pharmacy, Department of Pharmaceutical Sciences, 600 S. 43rd St, Philadelphia, Pennsylvania 19104, United States.

  [b] Present Address: Department of Pharmaceutical Industry, Hanoi University of Pharmacy, 13-15 Le Thanh Tong St., Cua Nam Ward, Hanoi, Vietnam. Zip code: 11021. https://orcid.org/0009-0005-6219-3228. Email: hoaltt@hup.edu.vn.

- Amos Abioye:

  [c] College of Pharmacy and Health Sciences, School of Pharmacy, Belmont University, 1900 Belmont Blvd., Tennessee 37212, USA. Email: amos.abioye@belmont.edu..

- Hai V. Nguyen:

  [d] Faculty of Pharmaceutical Chemistry and Technology, Hanoi University of Pharmacy, 13-15 Le Thanh Tong St., Cua Nam Ward, Hanoi, Vietnam. Zip code: 11021. https://orcid.org/0000-0003-4710-511X. Email: hainv@hup.edu.vn.

Contents

Supporting information for Tables....4

Table S1. Characteristics of SWCNT dispersions using CNTs oxidized for different periods. 4

Table S2. Weight loss of SWCNTs after oxidative acid treatment for different periods. 5

Table S3. Characteristics of CNT dispersions sonicated at different sonication power levels. 6

Table S4. Characteristics of SWCNT dispersions sonicated for different periods. 7

Table S5. Regression analysis of predicted models for the HDS....8

Table S6. Regression coefficients and p-values calculated from the HDS model. 9

Table S7. Regression analysis of predicted models for the PDI. 10

Table S8. Regression coefficients and p-values calculated from the PDI model. 11

Table S9. Regression analysis of predicted models for the ZP....12

Table S10. Regression coefficients and p-values calculated from the ZP model. 13

Table S11. Regression analysis of predicted models for the RAT....14

Table S12. Regression coefficients and p-values calculated from the RAT model. 15

Table S13. The optimal parameters for input factors and predicted responses of the SWCNT dispersion using the desirability function....16

Table S14. Observed responses of the optimal SWCNT dispersions using the desirability function and results of the optimization process validation. 17

Table S15. Validation results of the optimization process using the contour profiler....18

Supporting information for Figures....19

Figure S1. EDX spectra and elemental compositions of pristine SWCNTs and SWCNTs treated with hydrochloric acid and a mixture of H_2SO_4/HNO_3 1/3 for different days....19

Figure S2. Pareto plot analysis for the contribution of factors affecting HDS. 20

Figure S3. Prediction profiler plots for the HDS of aqueous SWCNT dispersions. 20

Figure S4. Two-factor interaction plots for the HDS of aqueous SWCNT dispersions. 20

Figure S5. Pareto plot analysis for the contribution of input factors affecting PDI. 21

Figure S6. Prediction profiler plots for the PDI of aqueous SWCNT dispersions. 21

Figure S7. Two-factor interaction plots for the PDI of aqueous SWCNT dispersions. 21

Figure S8. Pareto plot analysis for the contribution of factors affecting ZP. 22

Figure S9. Prediction profiler for the ZP of aqueous SWCNT dispersions. 22

Figure S10. Two-factor interaction plots for the ZP of aqueous SWCNT dispersions 22

Figure S11. Pareto plot analysis for the contribution of factors affecting RAT. 23

Figure S12. Prediction profiler for the RAT of aqueous SWCNT dispersions. 23

Figure S13. Two-factor interaction plots for the RAT of aqueous SWCNT dispersions. 23

Figure S14. Prediction profiler showing the optimized SWCNT dispersion with maximum desirability. 24

Figure S15. Contour profiler output showing the design space (white region) of SWCNT dispersions as a function of (A) oxidation time and sonication power; (B) oxidation time and sonication time, versus the responses: HDS (red region), PDI (green region), ZT (blue region), and RAT (yellow region). 24

Figure S16. The hydrodynamic particle size distribution (I) and zeta potential (II) of the pristine SWCNT dispersion. 25

Figure S17. The hydrodynamic particle size distribution (I) and zeta potential (II) of the optimal SWCNT dispersion. 26

Supporting information for Tables

Table S1. Characteristics of SWCNT dispersions using CNTs oxidized for different periods.

Oxidation time (day)	HDS (nm)	PDI	ZP (mV)	RAT
0	1469.7 ± 243.1	0.946 ± 0.070	-16.90 ± 1.90	0.058 ± 0.004
0.5	327.1 ± 8.1	0.448 ± 0.023	- 21.15 ± 1.91	0.078 ± 0.004
1	261.7 ± 7.6	0.415 ± 0.005	-50.19 ±2.29	0.094 ± 0.006
2	223.2 ± 5.0	0.387 ± 0.008	-57.13 ± 1.97	0.163 ± 0.006
3	189.6 ± 4.0	0.343 ± 0.007	-73.1 ± 1.48	0.242 ± 0.010

Table S2. Weight loss of SWCNTs after oxidative acid treatment for different periods.

**Sample**	**Weight loss** (%)				**Total of oxygenated functional groups (%)**
	25 – 130 (° C)	130 – 350 (° C)	350 – 500 (° C)	> 500 ° C	
Pristine CNT	1.241 ± 0.079	2.452 ± 0.145	1.555 ± 0.078	20.227 ± 0.530	4.007
o.CNT 2D	9.86 ± 0.447	9.25 ± 0.160	5.157 ± 0.539	32.241 ± 1.010	14.407
o.CNT 3D	5.766 ± 0.203	10.234 ± 0.387	5.43 ± 0.410	34.764 ± 0.921	15.664

(Raw material, HCl-treated CNTs oxidized for 2 and 3 days, denoted as pristine CNT, o.CNT 2D, o.CNT 3D, respectively)

Table S3. Characteristics of CNT dispersions sonicated at different sonication power levels.

Sonication Power (W)	HDS (nm)	PDI	ZP (mV)	RAT
25	374.2 ± 8.1	0.434 ± .008	-37.56 ± 1.29	0.065 ± 0.007
50	261.7 ± 7.6	0.415± 0.005	-39.63 ±2.29	0.094 ± 0.006
75	239.0 ± 5.6	0.366 ± 0.009	-53.00 ± 1.18	0.124 ± 0.005
100	212.1 ± 4.85	0.354± 0.007	-58.77± 0.327	0.133± 0.008

Table S4. Characteristics of SWCNT dispersions sonicated for different periods.

Sonication time (minutes)	HDS (nm)	PDI	ZP (mV)	RAT
3	280.8 ± 7.7	0.437 ± 0.011	-35.8 ± 1.2	0.081 ± 0.007
5	261.7 ± 7.6	0.415 ± 0.005	-39.63 ± 2.29	0.094 ± 0.006
10	217.5 ± 3.6	0.401 ± 0.009	- 47.87 ± 3.5	0.109 ± 0.004
15	196.8 ± 4.9	0.388 ± 0.007	-49. 61 ± 1.26	0.122 ± 0.008

Table S5. Regression analysis of predicted models for the HDS.

Summary of Fit

RSquare	0.985776
RSquare Adj	0.975431
Root Mean Square Error	6.768457
Mean of Response	220.2484
Observations (or Sum Wgts)	20

Analysis of Variance

Source	DF	Sum of Squares	Mean Square	F Ratio
Model	8	34923.375	4365.42	95.2899
Error	11	503.932	45.81	**Prob > F**
C. Total	19	35427.307		<.0001*

Lack Of Fit

Source	DF	Sum of Squares	Mean Square	F Ratio
Lack Of Fit	8	468.97551	58.6219	5.0310
Pure Error	3	34.95653	11.6522	**Prob > F**
Total Error	11	503.93204		0.1057
				**Max RSq**
				0.9990

Table S6. Regression coefficients and p-values calculated from the HDS model.

Term	Estimate	Std Error	t Ratio	Prob>|t|
Intercept	177.82846	3.432021	51.81	<.0001*
OXIDATION TIME(0.5,2.5)	-35.72986	1.827719	-19.55	<.0001*
SONICATION POWER(50,100)	-1.299888	1.881711	-0.69	0.5040
SONICATION TIME(5,15)	-0.105149	1.768369	-0.06	0.9537
OXIDATION TIME*SONICATION POWER	13.79861	2.084669	6.62	<.0001*
OXIDATION TIME*SONICATION TIME	11.21456	1.972642	5.69	0.0001*
SONICATION POWER*SONICATION TIME	6.5865609	1.997872	3.30	0.0071*
OXIDATION TIME*OXIDATION TIME	43.967961	3.633746	12.10	<.0001*
SONICATION TIME*SONICATION TIME	14.42292	3.874062	3.72	0.0034*

Table S7. Regression analysis of predicted models for the PDI.

Summary of Fit

RSquare	0.985527
RSquare Adj	0.975
Root Mean Square Error	0.006169
Mean of Response	0.36075
Observations (or Sum Wgts)	20

Analysis of Variance

Source	DF	Sum of Squares	Mean Square	F Ratio
Model	8	0.02850315	0.003563	93.6267
Error	11	0.00041860	0.000038	**Prob > F**
C. Total	19	0.02892175		<.0001*

Lack Of Fit

Source	DF	Sum of Squares	Mean Square	F Ratio
Lack Of Fit	8	0.00039260	0.000049	5.6625
Pure Error	3	0.00002600	8.667e-6	**Prob > F**
Total Error	11	0.00041860		0.0907
				**Max RSq**
				0.9991

Table S8. Regression coefficients and p-values calculated from the PDI model.

Term	Estimate	Std Error	t Ratio	Prob>|t|
Intercept	0.3287043	0.003128	105.09	<.0001*
OXIDATION TIME(0.5,2.5)	-0.024109	0.001666	-14.47	<.0001*
SONICATION POWER(50,100)	-0.006809	0.001715	-3.97	0.0022*
SONICATION TIME(5,15)	-0.00223	0.001612	-1.38	0.1938
OXIDATION TIME*SONICATION POWER	0.0247789	0.0019	13.04	<.0001*
OXIDATION TIME*SONICATION TIME	0.0154927	0.001798	8.62	<.0001*
SONICATION POWER*SONICATION TIME	0.0121173	0.001821	6.65	<.0001*
OXIDATION TIME*OXIDATION TIME	0.0304304	0.003312	9.19	<.0001*
SONICATION TIME*SONICATION TIME	0.0116174	0.003531	3.29	0.0072*

Table S9. Regression analysis of predicted models for the ZP.

Summary of Fit

RSquare	0.986319
RSquare Adj	0.976369
Root Mean Square Error	2.329294
Mean of Response	-50.0875
Observations (or Sum Wgts)	20

Analysis of Variance

Source	DF	Sum of Squares	Mean Square	F Ratio
Model	8	4302.5898	537.824	99.1268
Error	11	59.6817	5.426	**Prob > F**
C. Total	19	4362.2716		<.0001*

Lack Of Fit

Source	DF	Sum of Squares	Mean Square	F Ratio
Lack Of Fit	8	54.101462	6.76268	3.6357
Pure Error	3	5.580267	1.86009	**Prob > F**
Total Error	11	59.681729		0.1581
				**Max RSq**
				0.9987

Table S10. Regression coefficients and p-values calculated from the ZP model.

Term	Estimate	Std Error	t Ratio	Prob>|t|
Intercept	-58.62629	1.181094	-49.64	<.0001*
OXIDATION TIME(0.5,2.5)	-15.38479	0.628991	-24.46	<.0001*
SONICATION POWER(50,100)	-3.122549	0.647571	-4.82	0.0005*
SONICATION TIME(5,15)	-0.593866	0.608566	-0.98	0.3501
OXIDATION TIME*SONICATION POWER	4.4829835	0.717417	6.25	<.0001*
OXIDATION TIME*SONICATION TIME	2.203085	0.678864	3.25	0.0078*
SONICATION POWER*SONICATION TIME	1.362545	0.687547	1.98	0.0731
OXIDATION TIME*OXIDATION TIME	7.788178	1.250516	6.23	<.0001*
SONICATION TIME*SONICATION TIME	3.3612918	1.333218	2.52	0.0284*

Table S11. Regression analysis of predicted models for the RAT.

Summary of Fit				
RSquare				0.995429
RSquare Adj				0.992105
Root Mean Square Error				0.00388
Mean of Response				0.14165
Observations (or Sum Wgts)				20
**Analysis of Variance**				
**Source**	**DF**	**Sum of Squares**	**Mean Square**	**F Ratio**
Model	8	0.03606893	0.004509	299.4571
Error	11	0.00016562	0.000015	**Prob > F**
C. Total	19	0.03623455		<.0001*
**Lack Of Fit**				
**Source**	**DF**	**Sum of Squares**	**Mean Square**	**F Ratio**
Lack Of Fit	8	0.00010495	0.000013	0.6487
Pure Error	3	0.00006067	0.000020	**Prob > F**
Total Error	11	0.00016562		0.7228
				**Max RSq**
				0.9983

Table S12**.** Regression coefficients and p-values calculated from the RAT model.

**Term**	**Estimate**	**Std Error**	**t Ratio**	**Prob>|t|**
Intercept	0.1394964	0.001967	70.90	<.0001*
OXIDATION TIME(0.5,2.5)	0.0464905	0.001048	44.37	<.0001*
SONICATION POWER(50,100)	0.0161602	0.001079	14.98	<.0001*
SONICATION TIME(5,15)	0.0048452	0.001014	4.78	0.0006*
OXIDATION TIME*SONICATION POWER	0.001728	0.001195	1.45	0.1761
OXIDATION TIME*SONICATION TIME	0.0009509	0.001131	0.84	0.4183
SONICATION POWER*SONICATION TIME	0.0007009	0.001145	0.61	0.5530
OXIDATION TIME*OXIDATION TIME	0.0014193	0.002083	0.68	0.5098
SONICATION TIME*SONICATION TIME	0.0039094	0.002221	1.76	0.1061

Table S13. The optimal parameters for input factors and predicted responses of the SWCNT dispersion using the desirability function.

Input factors (X)	Predicted response (Y)
X_1 (oxidation time): 2 days	Y_1 (hydrodynamic SWCNT particle size, HDS): 165.4 (nm)
X_2 (sonication power): 50W	Y_2 (polydispersity index, PDI): 0.319
X_3 (sonication time): 10 minutes	Y_3 (zeta potential, ZP) -63.49 (mV)
	Y_4 (Raman ratio, RT): 0.146

Table S14. Observed responses of the optimal SWCNT dispersions using the desirability function and results of the optimization process validation.

Sample	Y_1	Y_2	Y_3	Y_4
1	171.9 ± 5.9	0.315 ± 0.017	-62.9 ± 1.9	0.154 ± 0.015
2	170.6 ± 3.5	0.317 ± 0.024	-66.7 ± 2.1	0.150 ± 0.008
3	160.3 ± 2.1	0.326 ± 0.015	-65.2 ± 0.13	0.143 ± 0.012
Mean	167.6 ± 6.3	0.319 ± 0.006	-64.93 ± 1.91	0.148 ± 0.005
Error (%)	1.31	0.00	2.22	1.35
p - value	0.6068	1.0000	0.3216	0.5601

Table S15. Validation results of the optimization process using the contour profiler.

Sample	Factors	Predicted responses	Observed responses	Error (%)	P - value
1	X_1: 1.25 days<br>X_2: 75 W<br>X_3: 10 min.	Y_1: 189.5	Y_1: 199.6; 180.1; 179.3	-1.70	0.6803
		Y_2: 0.337	Y_2: 0.341; 0.330; 0.352	1.17	0.5932
		Y_3: -54.29	Y_3: -51.88; -53.98; -50.71	-4.02	0.1593
		Y_4: 0.128	Y_4: 0.122; 0.129; 0.142	2.29	0.6596
2	X_1: 1.5 days<br>X_2: 65 W<br>X_3: 12 min.	Y_1: 179.6	Y_1: 180.5; 191.7; 185.3	3.35	0.1867
		Y_2: 0.331	Y_2: 0.331; 0.327; 0.343	0.80	0.1185
		Y_3: -57.30	Y_3: -58.7; -60.2; -58.5	3.10	0.0760
		Y_4: 0.135	Y_4: 0.129; 0.131; 0.134	-2.79	0.1276
3	X_1: 1.75 days<br>X_2: 90 W<br>X_3: 8 min.	Y_1: 172.6	Y_1: 175.6; 181.8; 177.2	3.14	0.0947
		Y_2: 0.323	Y_2: 0.341; 0.347; 0.330	4.81	0.0817
		Y_3: -62.96	Y_3: -65.65; -62.40; -67.88	3.60	0.2777
		Y_4: 0.160	Y_4: 0.153; 0.162; 0.169	0.83	0.8005

Supporting information for Figures

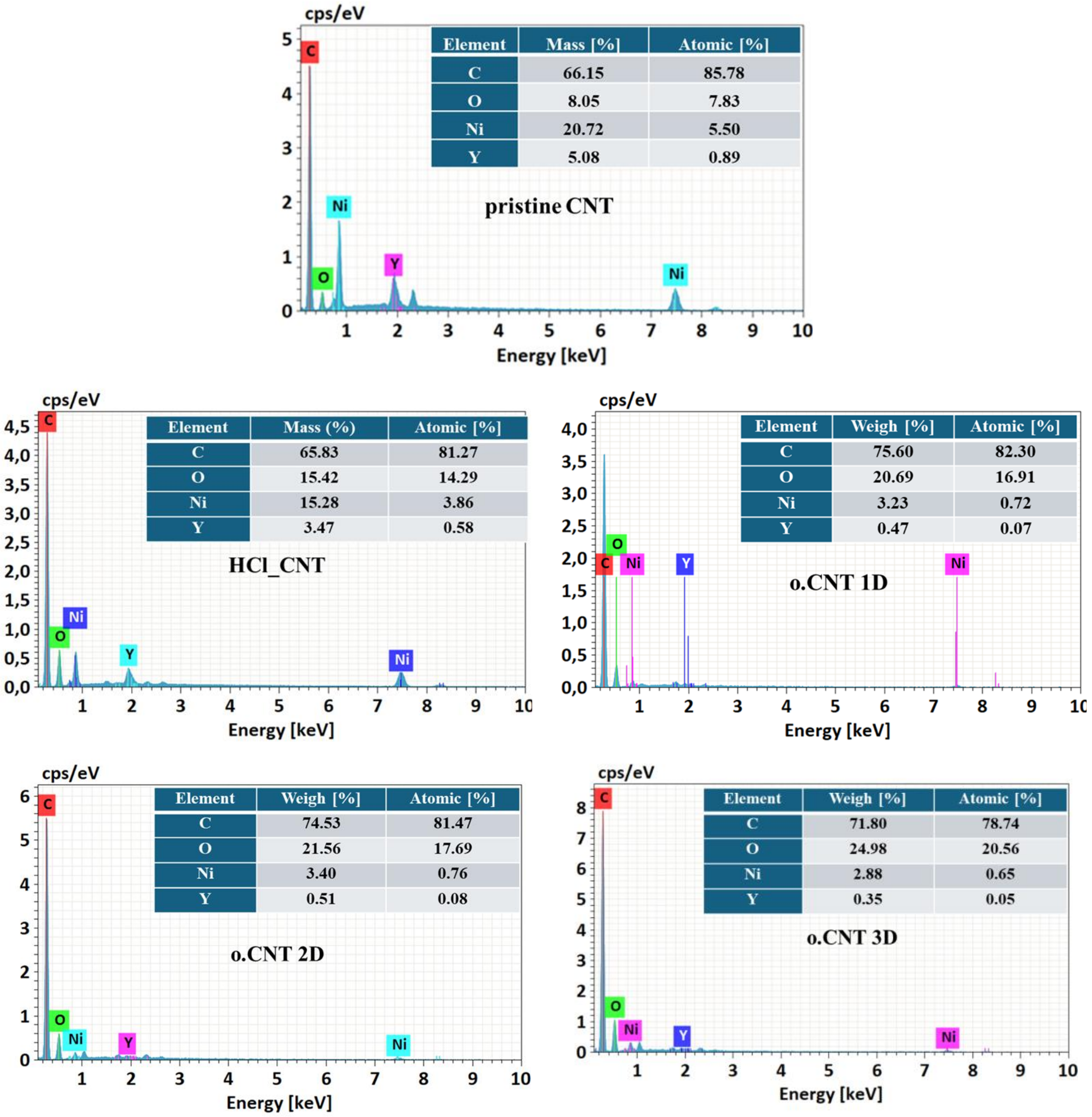

Figure S1. EDX spectra and elemental compositions of pristine SWCNTs and SWCNTs treated with hydrochloric acid and a mixture of H_2SO_4/HNO_3 1/3 for different days.

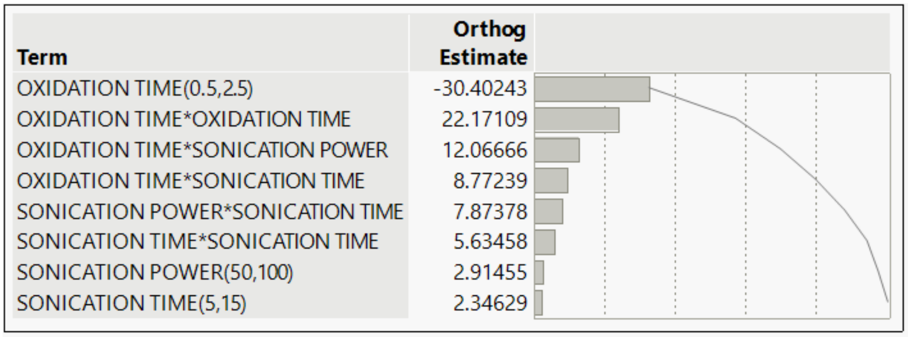

Figure S2. Pareto plot analysis for the contribution of factors affecting HDS.

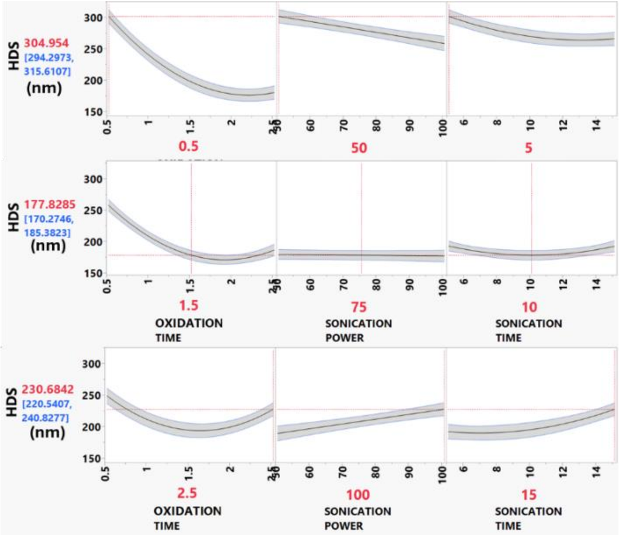

Figure S3. Prediction profiler plots for the HDS of aqueous SWCNT dispersions.

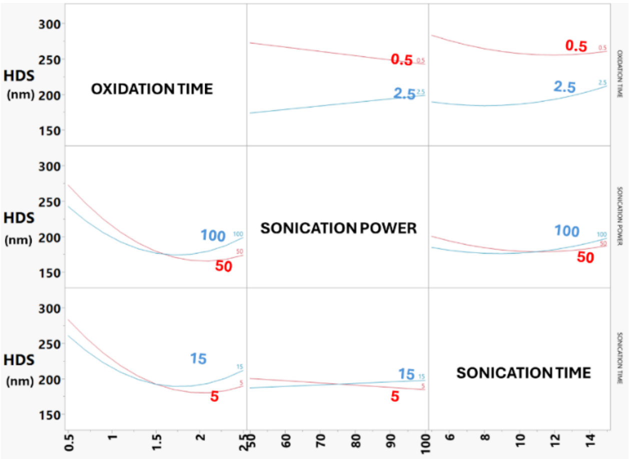

Figure S4. Two-factor interaction plots for the HDS of aqueous SWCNT dispersions.

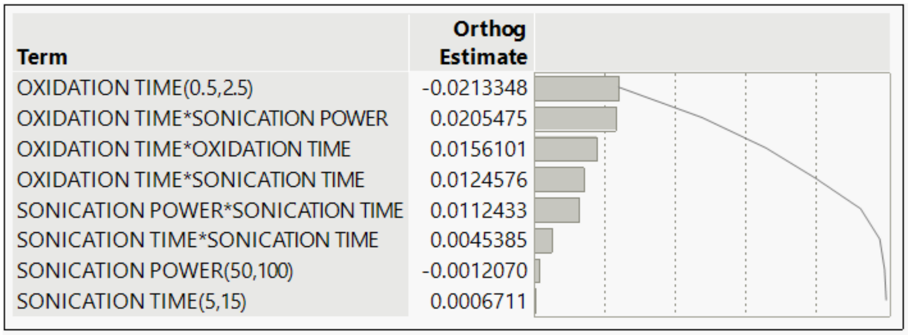

Term	Orthog Estimate
OXIDATION TIME(0.5,2.5)	-0.0213348
OXIDATION TIME*SONICATION POWER	0.0205475
OXIDATION TIME*OXIDATION TIME	0.0156101
OXIDATION TIME*SONICATION TIME	0.0124576
SONICATION POWER*SONICATION TIME	0.0112433
SONICATION TIME*SONICATION TIME	0.0045385
SONICATION POWER(50,100)	-0.0012070
SONICATION TIME(5,15)	0.0006711

Figure S5. Pareto plot analysis for the contribution of input factors affecting PDI.

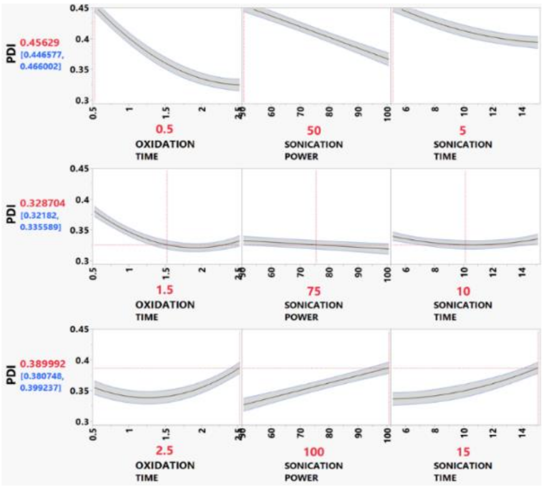

Figure S6. Prediction profiler plots for the PDI of aqueous SWCNT dispersions.

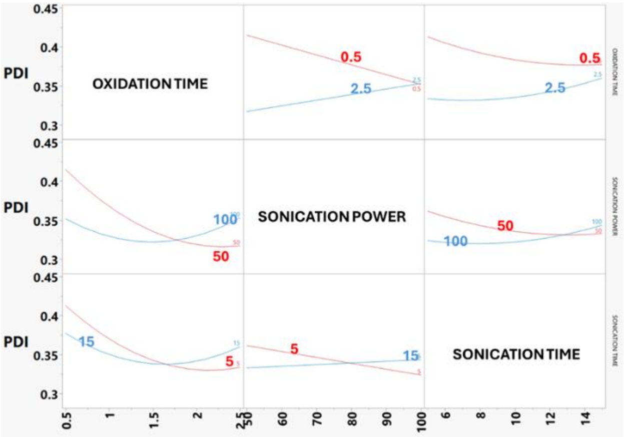

Figure S7. Two-factor interaction plots for the PDI of aqueous SWCNT dispersions.

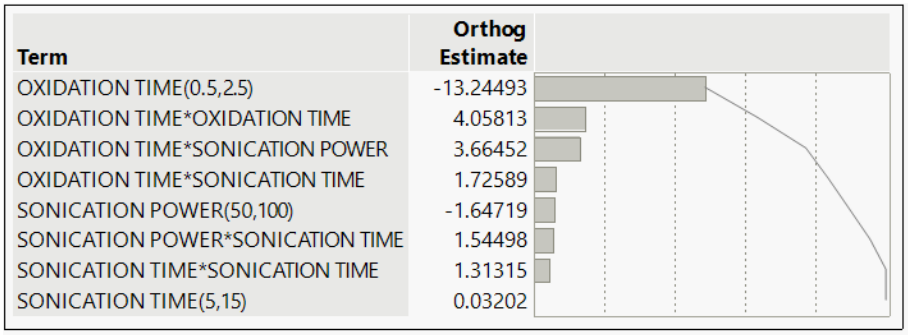

Figure S8. Pareto plot analysis for the contribution of factors affecting ZP.

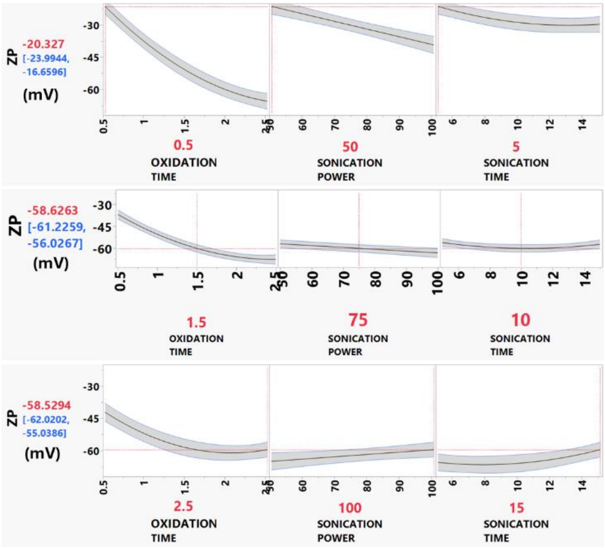

Figure S9. Prediction profiler for the ZP of aqueous SWCNT dispersions.

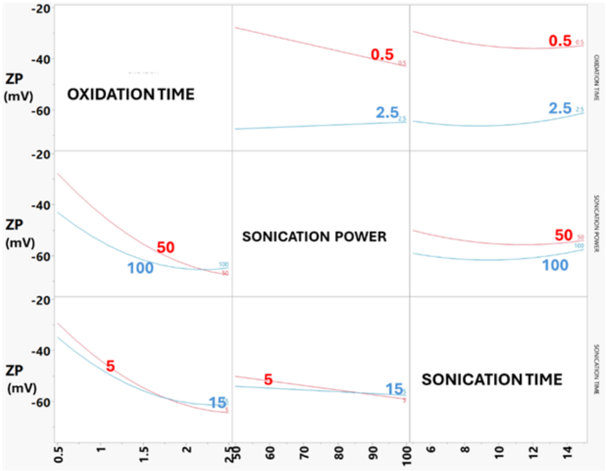

Figure S10. Two-factor interaction plots for the ZP of aqueous SWCNT dispersions.

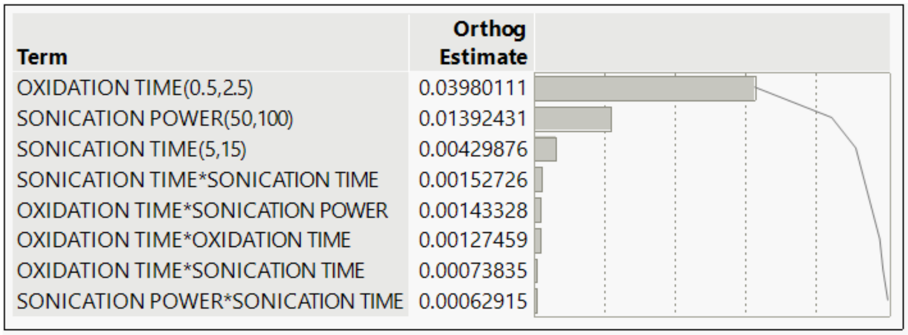

Figure S11. Pareto plot analysis for the contribution of factors affecting RAT.

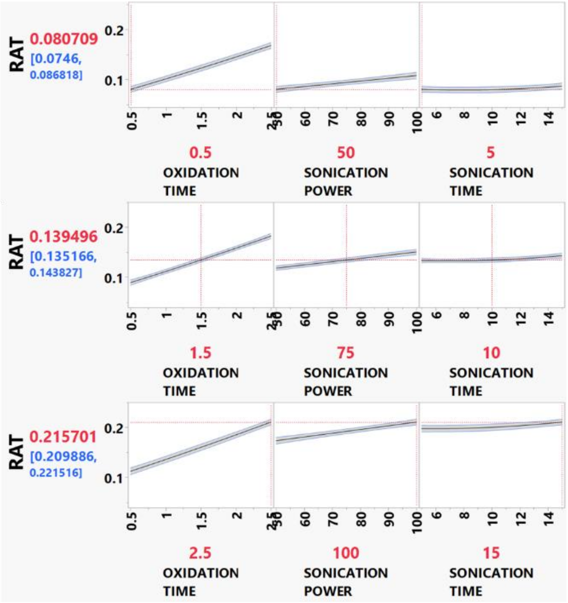

Figure S12. Prediction profiler for the RAT of aqueous SWCNT dispersions.

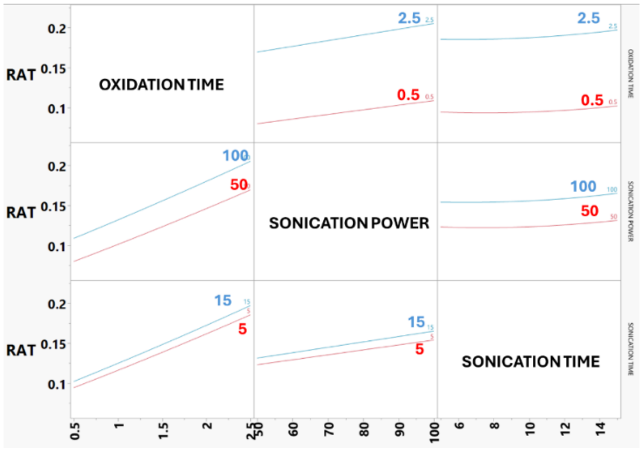

Figure S13. Two-factor interaction plots for the RAT of aqueous SWCNT dispersions.

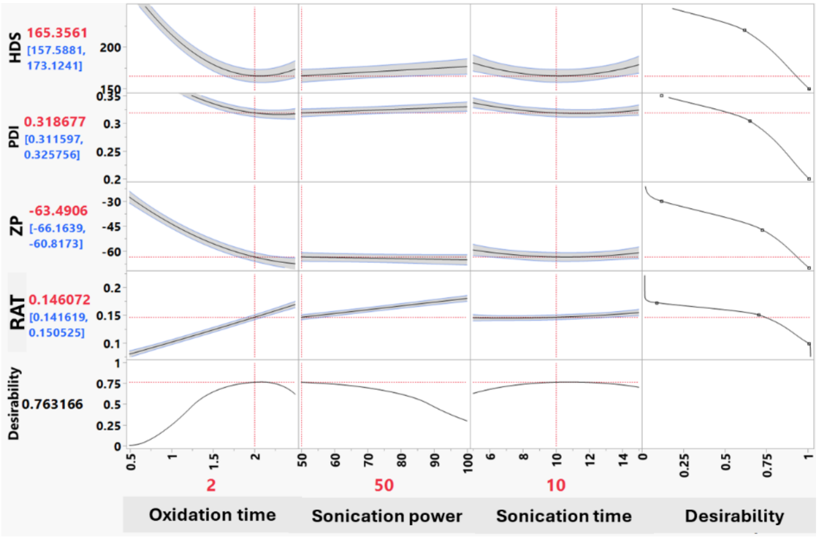

Figure S14. Prediction profiler showing the optimized SWCNT dispersion with maximum desirability.

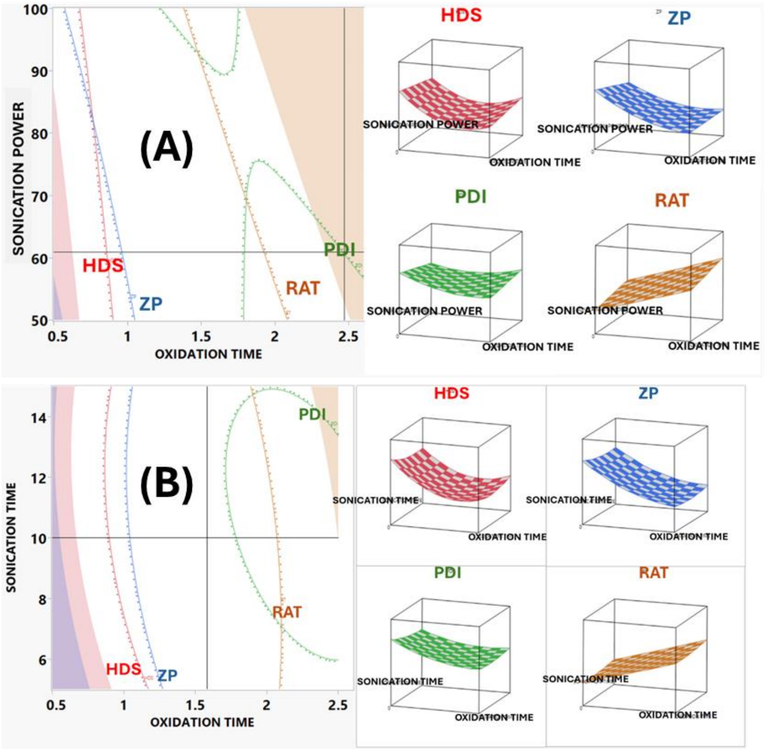

Figure S15. Contour profiler output showing the design space (white region) of aqueous SWCNT dispersions for (A) oxidation time and sonication power; (B) oxidation time and sonication time (B) versus the responses: HDS (red region), PDI (green region), ZT (blue region), and RAT (yellow region).

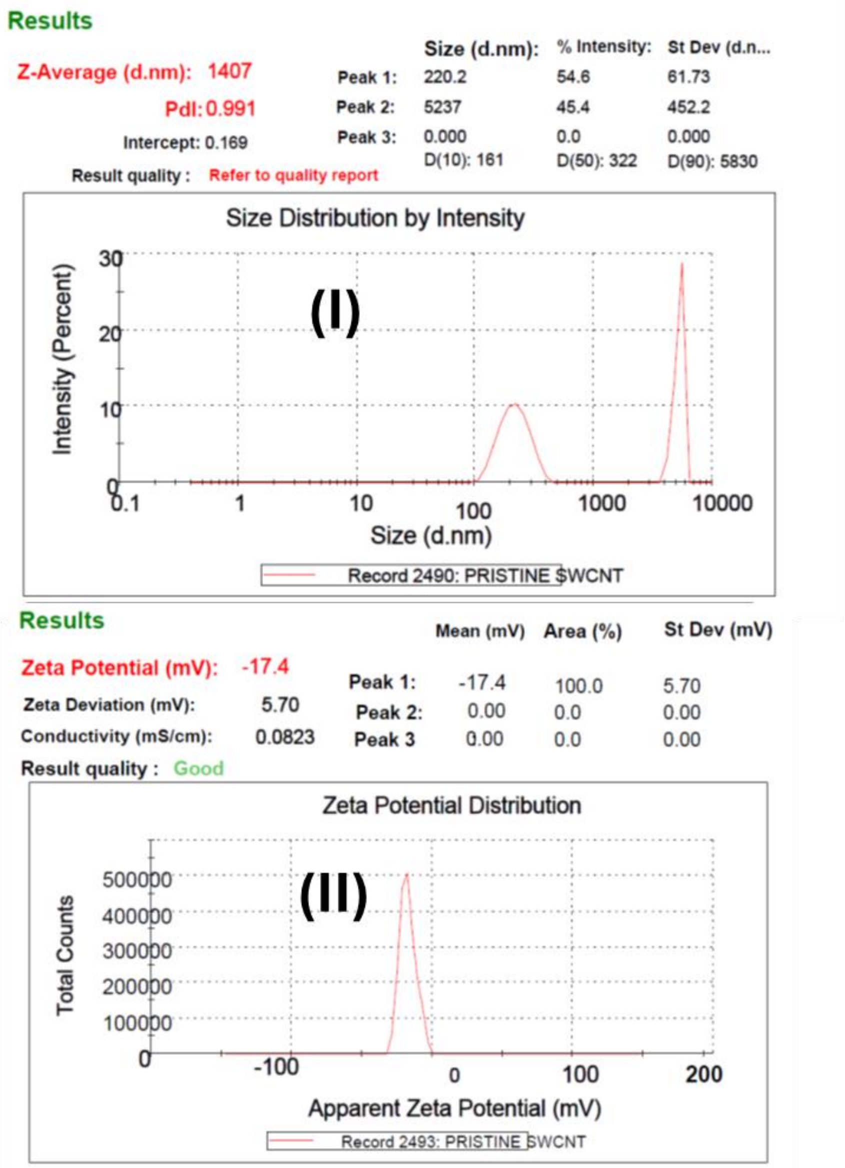

Figure S16. The hydrodynamic particle size distribution (I) and zeta potential (II) of the pristine SWCNT dispersion.

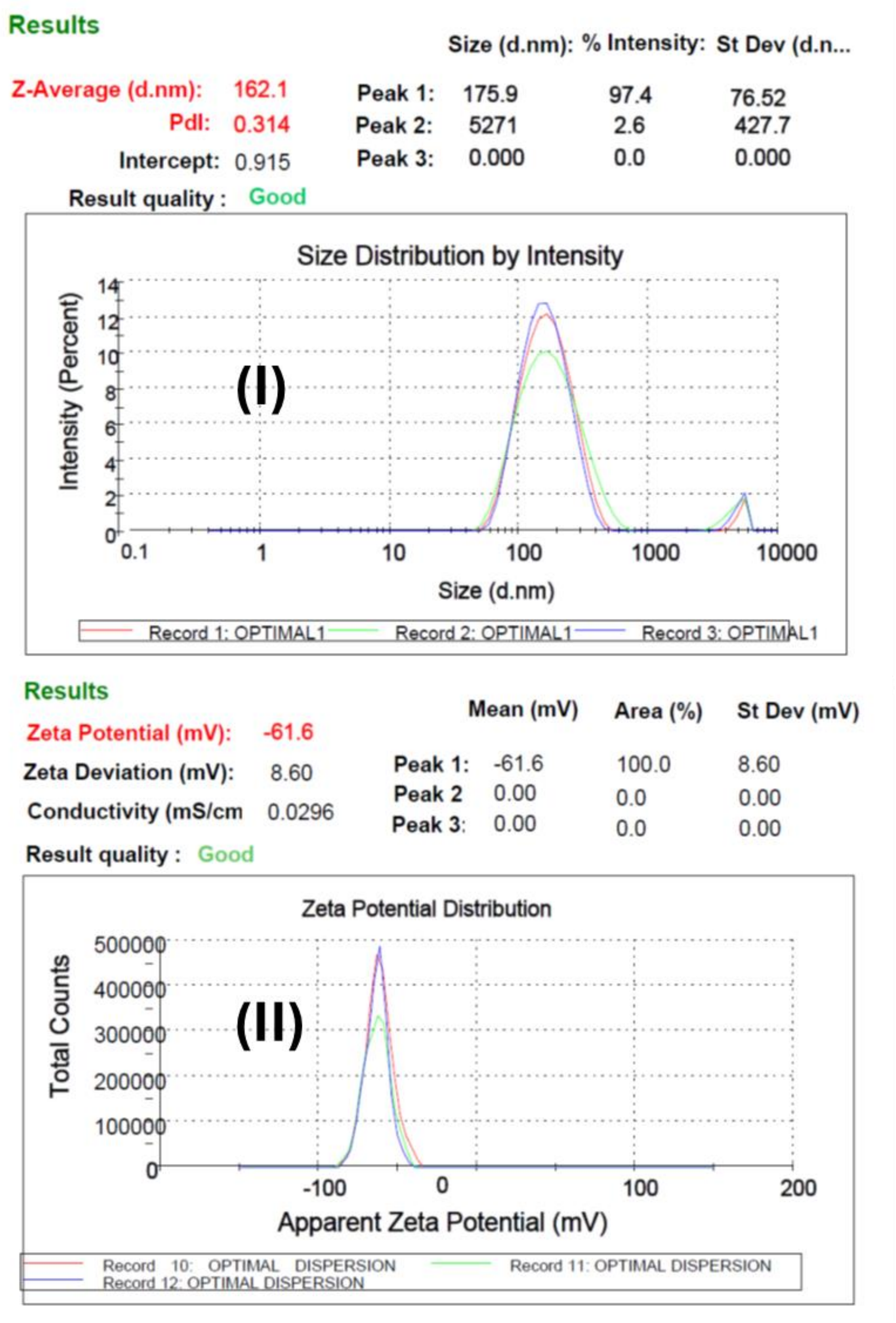

Figure S17. The hydrodynamic particle size distribution (I) and zeta potential (II) of the optimal SWCNT dispersion.
